# The role of viability selection in shaping short‐ and long‐term consequences of early‐life conditions: An experiment in the Alpine swift

**DOI:** 10.1111/1365-2656.70314

**Published:** 2026-07-13

**Authors:** Bashar Jarayseh, Giulia Masoero, Pierre Bize

**Affiliations:** ^1^ Department Biology II Ludwig‐Maximilians‐Universität München Planegg‐Martinsried Germany; ^2^ Swiss Ornithological Institute Sempach Switzerland; ^3^ Department of Ecology & Evolution University of Lausanne Lausanne Switzerland

**Keywords:** Apodiformes, brood size manipulation, life‐history trajectory, missing fraction, selective disappearance, sibling competition, silver‐spoon hypothesis, viability selection

## Abstract

1. Environmental conditions experienced early in life can shape growth, morphology and survival, and can have lasting effects on individuals that survive to adulthood. Understanding how early‐life conditions affect fitness components across the life cycle is therefore essential for predicting variation in life‐history trajectories and their ecological and evolutionary consequences.
2. We experimentally tested for short‐ and long‐term effects of early growth conditions by manipulating brood size in a wild population of the long‐lived Alpine swift *Tachymarptis melba*. Enlarging or reducing brood size alters sibling competition and per capita resource availability, with potential consequences on resource allocation between growth and self‐maintenance. We manipulated 45 pairs of broods between 2003 and 2006 and followed the offspring throughout their lives until 2019.
3. Brood enlargement reduced average growth and survival, while increasing phenotypic variability among nestlings. Nestlings from enlarged broods were smaller, fledged later, and had lower survival to fledging and recruitment. Recruitment probability increased with nestling size, indicating strong selection on developmental phenotype. Consequently, nestlings from enlarged broods that recruited were a non‐random subset of bigger, higher‐quality individuals, and morphological differences between treatments disappeared among adults. Despite lower recruitment, individuals from enlarged broods showed equal or higher lifetime reproductive output, primarily through more frequent breeding attempts. Age‐specific analyses showed that reproductive performance patterns reflected both within‐individual changes and selective disappearance among individuals.
4. We discuss how early‐life conditions shape life‐history trajectories through both developmental effects and selection acting before adulthood, arguing that apparent advantages in adult performance among individuals from harsher developmental environments arise from viability selection rather than beneficial effects of early‐life stress. Our findings show how missing‐fraction problems can bias inferences about fitness when early‐life mortality is ignored, highlighting the need to integrate early survival when evaluating the lifelong consequences of developmental environments.

## Graphical abstract

In an experimental brood‐size manipulation in Alpine swifts, smaller nestlings were selectively lost during growth, eliminating treatment differences in adult size, yet individuals from enlarged broods showed higher reproductive performance.

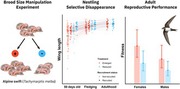

## 1 INTRODUCTION

Stressful environmental conditions, such as food shortage, parasitism or competition, experienced early in life can have strong short‐term effects on growth, morphology and survival (Boag, 1987; Dantzer et al., 2013; Fitze et al., 2004). Importantly, early‐life conditions can also have lasting effects on the phenotype and life‐history trajectories of individuals that survive to adulthood (Boag, 1987; De Kogel & Prijs, 1996; Fitze et al., 2004; Schluter & Gustafsson, 1993). Such long‐term effects are referred to as ‘silver‐spoon’ effects, when individuals that were raised under favourable conditions achieve higher fitness in adulthood than those raised in poorer conditions (Grafen, 1988; Lindström, 1999; Monaghan, 2007; van de Pol et al., 2006; Wilson & Burley, 2024). Understanding how early developmental conditions shape fitness components across the life cycle and generate variation in life‐history trajectories is therefore central to predicting ecological and evolutionary dynamics. Such variation determines how populations respond to selection and ultimately reshapes their adaptive potential (Lindström, 1999).

The short‐term effects of adverse environmental conditions encountered early in life are well studied, ranging from changes in offspring physiology and behaviour to morphology and survival up to independence (Burness et al., 2000; Dijkstra et al., 1990; Fitze et al., 2004; Kogel, 1997; Sepers et al., 2021). Since organisms depend on limited resources to ‘build’ their phenotype, early‐life constraints are typically mediated by trade‐offs in resource allocation between combinations of molecular and physiological functions or tissues, some of which are more strongly protected than others during development (Boersma & Wit, 1997; Metcalfe & Monaghan, 2001). Such constraints often generate variation in developmental trajectories, with individuals differing in growth rates, body conditions and allocation strategies (Boonekamp et al., 2018; Dmitriew, 2011; Stearns, 1992). Developmental plasticity can buffer these effects in the short term by allowing organisms to survive through a detrimental time period in early‐life, and sometimes even to catch up after a bad start (Bize, Metcalfe, & Roulin, 2006; Boersma & Wit, 1997; Hector & Nakagawa, 2012; Kogel, 1997), but these adjustments often involve trade‐offs that can lead to long‐term fitness costs (Johnsson & Bohlin, 2006; Mangel & Munch, 2005; Metcalfe & Monaghan, 2001). Importantly, this variation in developmental trajectories may translate into differential survival, thereby shaping the distribution of phenotypes that persist to adulthood.

Hence, when examining long‐term consequences of early‐life conditions in natural populations, it is essential to account for non‐random mortality during development. Individuals most strongly affected by adverse conditions may fail to survive to adulthood, resulting in the recruitment of a subset of individuals biased towards higher genetic or phenotypic quality (the so‐called ‘viability selection’ hypothesis; Grafen, 1988; Hadfield, 2008). Consequently, individuals originating from harsh rearing conditions may represent a non‐random subset of survivors skewed towards traits that enhance resilience or competitive ability (Hadfield, 2008; Mittell & Morrissey, 2024; Mojica & Kelly, 2010). The viability selection can also lead to ‘selective appearance’ or ‘selective disappearance’, where individuals are selectively included in, or excluded from, older age classes through non‐random survival. Such selective inclusion or exclusion can generate misleading patterns when only adult individuals are considered, creating the impression that individuals from poor early environments perform as well as, or better than, those from favourable conditions (Drake et al., 2025). More generally, such ‘missing fraction’ problems arise because individuals that do not survive to express adult traits are excluded from analyses, biasing inferences about the effect of early‐life conditions on fitness (Grafen, 1988; Mojica & Kelly, 2010).

Alternative explanations to viability selection favouring fitter phenotypes have been proposed to explain positive effects of early‐life stress on later performance. First, the ‘predictive adaptive response’ hypothesis, or match–mismatch effect (Gluckman & Hanson, 2004; Monaghan, 2007), posits that early‐life cues shape development to match the expected adult environment, so that individuals born under harsh conditions may perform best in similarly harsh adult conditions. Second, the ‘hormesis’ hypothesis proposes that mild early‐life stress primes physiological responses, enhancing resilience and coping upon re‐exposure to stress in adulthood (Calabrese & Baldwin, 2002; Costantini, 2010). Although conceptually appealing, this dose‐dependent hypothesis still lacks strong empirical support within an eco‐evolutionary framework and therefore remains debated (Costantini, 2019; Forbes, 2000).

Experimental manipulations are a powerful approach to isolate the causal effects of early growth conditions in natural populations (Krist, 2011; Monaghan, 2007). In bird species where growing offspring rely entirely on their parents for resources (i.e. altricial and semi‐precocial nestlings), one approach frequently used to create contrasting rearing environments is to manipulate the number of siblings per brood. Increasing the number of siblings increases competition and reduces per capita resource availability, whereas reducing brood size has the opposite effect. As a result, nestlings in enlarged broods typically grow more slowly and may experience higher mortality than those in reduced broods (Burness et al., 2000; Cornelius Ruhs et al., 2020; Cucco & Malacarne, 1996; Ilmonen et al., 2003; Roulin et al., 2008; Tschirren et al., 2009; Vitousek et al., 2017). However, variation in parental quality may also influence the extent of these differences among broods (Descamps et al., 2016). Despite extensive evidence for short‐term effects, the long‐term effects of being reared in an enlarged versus reduced brood on the same individuals in adulthood remain little investigated, especially in wild populations where longitudinal data are difficult to obtain (Cam & Aubry, 2011). Brood size manipulation experiments in captive zebra finches *Taeniopygia guttata* showed that individuals reared in reduced broods were heavier during growth and in adulthood, and had lower metabolic rates and mortality in adulthood (Briga et al., 2017; De Kogel & Prijs, 1996; Kogel, 1997; Reichert et al., 2015; Verhulst et al., 2006). To our knowledge, only one study reported life‐long effects of experimental brood size manipulation in a wild bird, showing silver‐spoon effects in female collared flycatchers *Ficedula albicollis* reared in reduced broods (Spagopoulou et al., 2020).

In this study, we investigated the short‐ and long‐term consequences of early growth conditions using a brood size manipulation experiment in a wild population of the long‐lived Alpine swift *Tachymarptis melba*. Specifically, we asked (1) how brood size affects growth and survival during development, and (2) how these early conditions influence adult morphology, reproductive success and lifespan. Following results of previous experiments manipulating brood size in birds, we predicted that offspring in enlarged broods would grow more slowly, reach a smaller or similar size at fledging depending on whether they can catch‐up for their poor start (Bize et al., 2003; Bize, Metcalfe, & Roulin, 2006) and suffer higher mortality before fledging or recruitment. Long‐term effects are less straightforward, as different processes can generate contrasting outcomes. Under the silver‐spoon hypothesis, individuals reared in poor conditions are expected to show reduced performance later in life, such as smaller adult size and lower reproductive success. If strong viability selection occurs during development, individuals surviving early adverse conditions (i.e., enlarged broods) may later display worse, neutral or even better performance, depending on which traits are under selection and how these traits are related to performance later in life. For example, if smaller individuals are more likely to survive early adverse conditions and remain small as adults, they may later perform less well, whereas if larger individuals are more likely to survive such conditions and remain large as adults, they may later perform better in life. We also tested for predictive adaptive responses by exploring whether effects of early growth environment (i.e. treatment) on reproductive performance were year‐dependant. Following the predictive adaptive response hypothesis (Gluckman & Hanson, 2004; Monaghan, 2007), we predicted that individuals from enlarged broods (experiencing an adverse early environment) would achieve higher reproductive success than those from reduced broods in harsh years, whereas the opposite pattern would be expected in mild years. As Alpine swifts show little sexual dimorphism (Dumas et al., 2025) and provide biparental care (Arn, 1960), we did not make strong a priori predictions about sex‐specific responses (however, for evidence of female‐biased mortality in response to parasitism, see Bize et al., 2005). Nevertheless, sex‐specific effects of early‐life conditions have been reported in other wild vertebrates, with one sex sometimes showing stronger or more variable fitness responses (Appleby et al., 1997; Drake et al., 2025; Reid et al., 2003). Finally, previous studies on this species suggest a hierarchy in tissue allocation during development under resource limitation, with body mass prioritized over structural traits such as sternum and wing length (Bize, Metcalfe, & Roulin, 2006; Masoero et al., 2024). At the nestling stage, we therefore expected treatment effects to be more pronounced for traits less closely linked to survival, such as sternum and wing length.

## 2 MATERIALS AND METHODS

### 2.1 Study species and data collection

The Alpine swift is a migratory, colonial bird breeding in holes in cliffs and under roofs of tall buildings. It is socially monogamous and reproduces in colonies ranging from a few pairs to several hundred pairs. In Switzerland, these long‐distance migrants arrive from their non‐breeding grounds in Western Africa in early April but do not start breeding until May and June; they leave their breeding ground in September (Meier et al., 2020). Females lay a single clutch of one to four eggs per year. Then, both sexes share incubation of the eggs for 18 days and feed their offspring until fledging, which occurs around 55 days after hatching (range 50–76) (Bize et al., 2004; Masoero et al., 2024). Alpine swifts reach sexual maturity at 2–3 years of age, and recruited birds do not disperse after starting to breed in a colony (Bize et al., 2017). This long‐lived species has an average adult lifespan of 7 years, and a maximum longevity record of 26 years (Fransson et al., 2010; Moullec et al., 2023).

Data were collected between 2003 and 2025 foremost in an urban colony of Alpine swifts of *ca*. 100 breeding pairs located under the roof of a clock tower in Biel, Switzerland, where the brood size manipulation was carried out (see Section 2.2). Data were also collected in a neighbouring urban colony of *ca*. 50 breeding pairs in Solothurn, located 26 km away, where nestlings fledged from Biel are frequently recruited as breeders. Each year, both colonies were regularly visited to record laying dates, clutch sizes and brood sizes at hatching and fledging, and breeders were caught by hand while sitting on eggs or hatchlings. Non‐breeding birds were caught while prospecting in the colony during the day, or during night captures at the start or end of the breeding season when all the doors to the colony are closed and every bird present in the colony is captured. At capture, for both nestlings and adults we measured their flattened wing length with a ruler to the nearest 1 mm, sternum size with a calliper to the nearest 0.1 mm, and body mass with a digital scale to the nearest 0.1 g. Adult structural traits are highly repeatable in this population, with annual repeatability of 0.85 for wing length and 0.95 for sternum length (see Dumas et al., 2025; Masoero et al., 2024).

### 2.2 Brood size manipulation experiment

To manipulate early growth conditions, we manipulated brood sizes in 45 pairs of nests (total is 90 nests and 223 nestlings; Table S1) matched by clutch size, hatching date, and brood size at hatching in 2003 (*N* = 16 pairs of nests), 2004 (*N* = 14) and 2006 (*N* = 15). For each pair of nests, two hatchlings (i.e. reduced broods) were exchanged for one hatchling (i.e. enlarged broods) 5 days after hatching in 2003 and 2 days after hatching in 2004 and 2006. Prior to manipulation, nests contained two to three hatchlings, thus the brood size after manipulation stayed within the natural range of one to four hatchlings. Care was taken to use different experimental breeding pairs in the different years to avoid pseudoreplication. Note, however, that because we did not include an unmanipulated control group or a broader gradient of treatment levels, our design limits our ability to identify potential non‐linear or threshold effects of developmental conditions.

Hatchlings were individually marked at hatching with nontoxic permanent colour markers prior to cross‐fostering, and at 10 days of age, they were ringed with a unique numbered aluminium ring. Nestlings' wing length, sternum length and body mass were measured regularly throughout from hatching until fledging (every 5–10 days until 50 days and then every 2 days up to fledging) as described in Section 2.1. Since there is no clear phenotypic difference between sexes at the nestling (or adult) stage, individuals were genetically sexed from blood cell DNA, using polymerase chain reaction (PCR) amplification of the CHD (chromo‐helicase‐DNA‐binding) genes (Griffiths et al., 1998). Sex was unknown for 10 nestlings that died early.

Further information on the experimental setup can be found in Bize et al. (2010); Bize, Gasparini, et al. (2006); Bize and Roulin (2006); and Roulin et al. (2008), along with partial information on short‐term experimental effects on nestling growth and survival. The analyses presented in this study on short‐term effects are, however, exhaustive, including all 3 years of the brood size manipulation and describing in detail the effects on nestling survival, growth, and size at fledging. Analyses of long‐term consequences are new. All procedures and methods involving animal measurements and ringing were reviewed and approved by the Swiss Federal Agency of Environment, Forests and Landscapes (project number 72[BE, SO]). The brood size manipulation was done under the legal authority of the Swiss Veterinary Office (permits 54/02 and 56/05).

### 2.3 Monitoring of the long‐term consequences

We investigated the long‐term consequences of being raised in enlarged or reduced broods in 2003, 2004 or 2006, by following the fate of offspring fledged from those experimental nests, from recruitment (i.e. recaptured as adults) until they were last seen in the population in 2019. Young non‐breeding Alpine swifts are often prospecting several colonies before settling for breeding. Fledglings in Biel are primarily recruited locally (i.e. in Biel and Solothurn), and recruits do not disperse after starting to breed in a colony (Bize et al., 2017). As the annual recapture rate of breeders in those two colonies is virtually 1 (Bize, Gasparini, et al., 2006), we estimated individual age at first reproduction as the age (in years) when an individual (recruit) was first observed breeding in the focal colonies, and lifespan as the age in years when they were last observed in the focal colonies. We estimated reproductive success (both annual and lifetime) as the number of eggs, hatchlings, fledglings and recruits produced, in addition to hatching rate and fledging rate. Birds that were caught roosting in the colonies at adulthood, but were never seen breeding, were included in the analyses of survival up to adulthood (recruitment), but they were excluded from the analyses of age at first reproduction, lifespan and reproductive success.

### 2.4 Statistical analyses

All statistical analyses were performed in R version 4.5.2 (R Core Team, 2025). Generalized additive models (GAMs) were fitted using the ‘gam()’ function from the ‘mgcv’ package (Wood, 2011). Linear mixed models (LMMs) and generalized linear mixed models (GLMMs) were fitted using the ‘glmmTMB’ package (Brooks et al., 2017; McGillycuddy et al., 2025). Significance of fixed effects was assessed using likelihood ratio tests from the ‘drop1’ function, and non‐supported interaction terms were removed from the final models. Estimated marginal means and pairwise contrasts were obtained using the ‘emmeans’ package (Lenth, 2024), based on the fitted mixed models and thus accounting for the included random effects.

LMMs and GLMMs included natal treatment (2‐level factor: Reduced, Enlarged), sex (2‐level factor: Male, Female), their interaction, and hatching year (3‐level factor: 2003, 2004, 2006) as fixed effects, and nest identity as a random intercept to account for non‐independence among nestlings from the same nest. Any deviations from this general structure are described below. Model error distributions were chosen according to the response variable: Gaussian for continuous traits (e.g. mass, wing and sternum length, fledging age), binomial for binary responses (e.g. survival, recruitment), beta‐binomial for success proportions (e.g. hatching success, fledging success), negative binomial for overdispersed count data and Conway–Maxwell–Poisson for underdispersed count data.

#### 2.4.1 Nestlings' phenotype and survival

To examine nestling growth trajectories in body mass, wing length and sternum length, we fitted GAMs with Gaussian error distributions. The analysis was restricted to nestlings that survived to at least 50 days of age to ensure comparable longitudinal data, and all repeated measurements for these individuals were included in the model. Treatment, sex and hatching year were included as fixed effects, and treatment‐specific smooth functions of age (days since hatching) were fitted to test whether growth trajectories differed between treatments. Nest identity was included as a random effect to account for non‐independence among nestlings from the same nest, and individual identity was included as a factor‐smooth interaction with age to account for repeated measures of the same nestlings and to allow individual‐specific deviations from the treatment‐level growth curve. Models were fitted using restricted maximum likelihood with shrinkage selection to avoid overfitting. Age‐specific differences between treatments were compared by calculating pairwise differences between treatment‐specific smooths of age (function ‘difference_smooths’, package ‘gratia’; Simpson, 2024). Differences were considered significant if the 95% confidence intervals did not overlap zero.

Treatment effects on morphological traits (body mass, wing length and sternum length) at specific developmental stages (day 30, day 50 and fledging) were analysed using Gaussian mixed models following the general model structure described above. To test whether brood enlargement affected phenotypic variability, residual dispersion was modelled using the dispersion component of Gaussian mixed models in ‘glmmTMB’, with treatment and sex as predictors. From these models, we calculated standard deviation ratios (Enlarged/Reduced) and their 95% confidence intervals.

Nestling survival was analysed using two complementary approaches. First, survival to fledging was analysed using a binomial GLMM. Second, age‐specific mortality during the nestling period was analysed using a Cox proportional hazards model (‘survival’ package; Therneau, 2024; Therneau & Grambsch, 2000), including treatment, sex and hatching year as fixed effects and nest identity as a shared frailty term to account for non‐independence among nestlings from the same nest. Fledging age was analysed using Gaussian mixed models with the general model structure described above.

#### 2.4.2 Recruitment

Recruitment was defined as whether an individual later appeared as an adult in the monitored colonies. Recruitment probability was analysed using binomial GLMMs with the general model structure described above. We analysed recruitment both for all hatchlings involved at the start of the brood size manipulation experiment and by considering only those that reached fledging to distinguish treatment effects before and after the fledging stage. We also tested if recruitment is affected by the fledging age using linear and quadratic terms of fledging age to allow for non‐linear relationships.

To examine whether recruitment depended on nestling phenotype and therefore to estimate viability selection on pre‐fledging morphology, we modeled recruitment probability as a function of morphological traits (body mass, wing length and sternum length) measured before fledging (day 50). Because recruitment reflects survival to adulthood, this analysis tests whether pre‐fledging morphology was associated with subsequent viability. We used binomial GLMMs including the morphological trait, treatment, their interaction, sex and hatching year as fixed effects. We also compared morphological traits between recruited and non‐recruited individuals at day 50 and at fledging using Gaussian mixed models including recruitment status (binary), treatment, their interaction, sex and hatching year as fixed effects, with nest identity as a random intercept. Morphology of recruited individuals at adulthood was analyzed using similar Gaussian mixed models (without recruitment status as a predictor). For adult body mass model, we additionally included the hour of capture and the day of capture relative to May 1st to control the large daily and seasonal variation in body mass in this species (Dumas et al., 2024).

#### 2.4.3 Adult life‐history traits

Adult life‐history traits were analysed for individuals that recruited into the breeding population. Age at first reproduction, lifespan and reproductive lifespan were analysed using Gaussian models with treatment, sex, their interaction and hatching year as fixed effects. Lifetime reproductive success (total eggs, hatchlings and fledglings) and the number of breeding attempts were analysed using GLMMs with negative binomial error distributions. A Poisson distribution was used for the number of recruits because the negative binomial model failed to converge. To distinguish between differences in reproductive frequency and per‐attempt output, an additional set of models included the log of the number of breeding attempts as an offset.

Age‐specific reproductive performance was analysed using repeated breeding records for each individual. Annual clutch size, number of hatchlings and number of fledglings were analysed using Conway–Maxwell–Poisson models, whereas hatching success and fledging success were analysed using beta‐binomial models. To separate within‐individual changes from among‐individual differences, age was decomposed into two components: a within‐individual component (age centred on each individual's mean age) and a between‐individual component (each individual's mean age across all observations) (van de Pol & Wright, 2009). Both components were standardized and included simultaneously in the models, along with their two‐way interactions with treatment and sex. Hatching year was included as a cohort control and individual identity as a random intercept.

Finally, we tested for predictive adaptive responses in reproductive success by exploring whether effects of early growth environment (i.e. treatment) on reproductive performance were year‐dependent. To quantify annual environmental conditions independently of the experimental manipulation, we used the full colony dataset to calculate an annual index of environmental quality. This index was defined as the colony‐wide mean nestling survival in a given year, measured as the proportion of offspring surviving from hatching to fledging, then standardized so that a negative index indicates worse than average conditions and a positive index better than average conditions. We then tested for treatment by annual environment quality interactions in the annual reproductive models described above.

## 3 RESULTS

### 3.1 Phenotype and survival at the nestling stage

**FIGURE 1:**
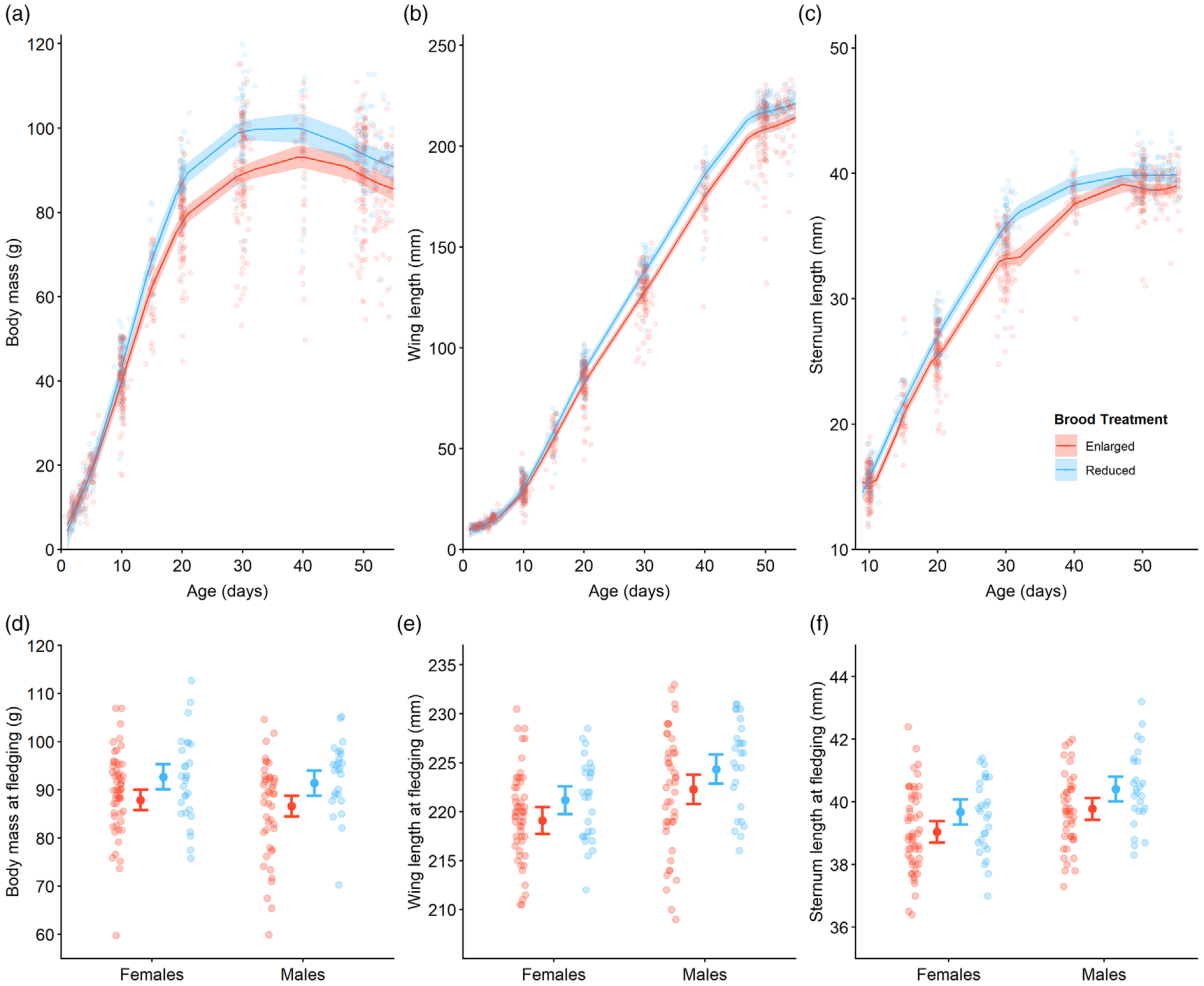
Growth trajectories of nestling Alpine swifts from hatching until day 55, for body mass (g; a), wing length (mm; b), and sternum length (mm; c) as functions of age, as well as size at fledging for body mass (g; d), wing length (mm; e), and sternum length (mm; f). Nestlings were reared in experimentally reduced (blue) or enlarged broods (red). In panels (a) to (c), the solid lines are model‐predicted growth trajectories, and the shaded areas are the corresponding 95% confidence intervals. In panels (d) to (f), the error bars are the mean and the 95% confidence intervals of the model predictions.

**FIGURE 2:**
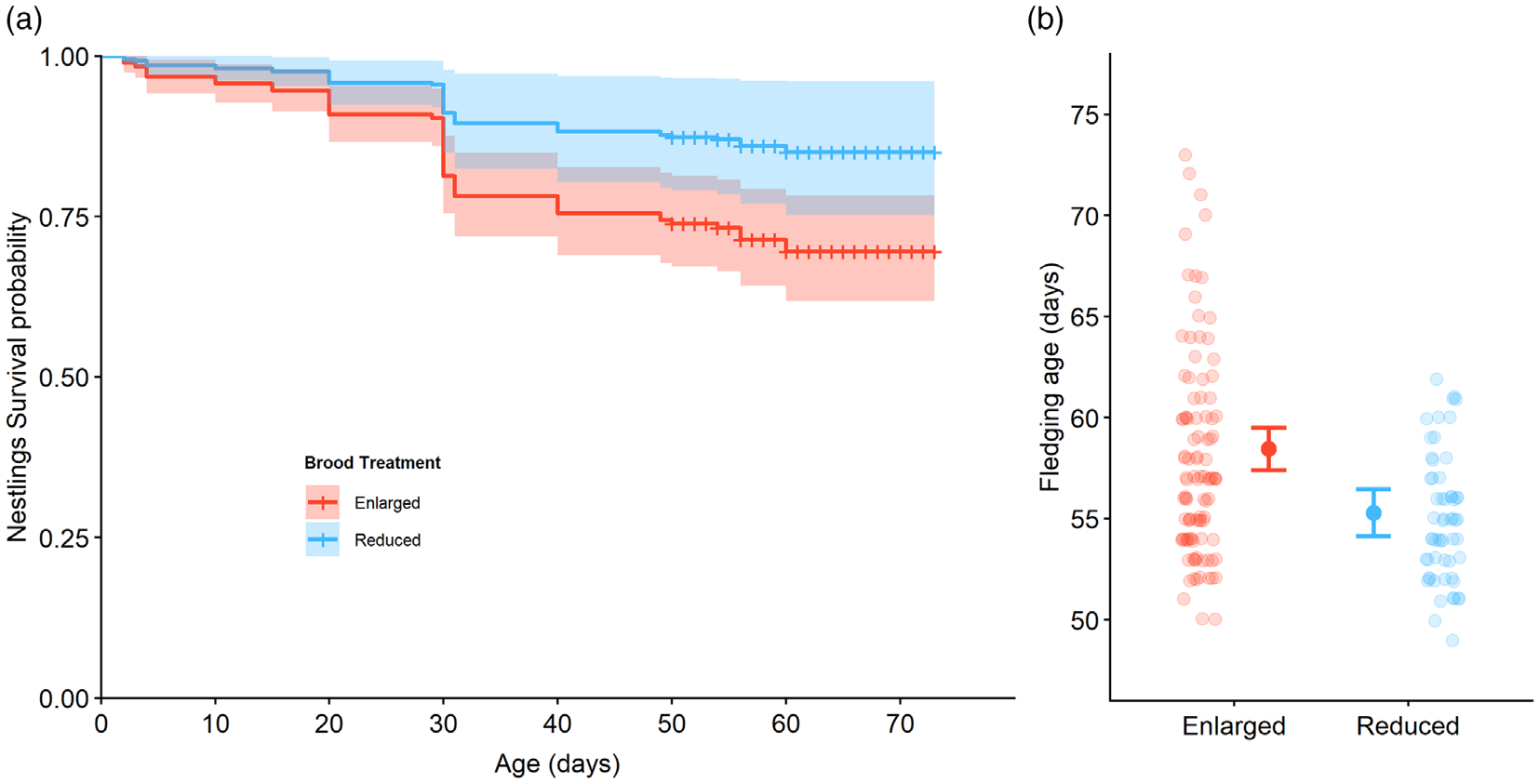
(a) Adjusted survival curves from a Cox proportional hazards model showing nestling survival probability by brood size treatment. Time represents days since hatching. Shaded areas indicate the 95% confidence intervals. Fledged individuals were right‐censored at the day following their last observation alive. (b) Age at fledging of Alpine swift nestlings from experimentally enlarged and reduced broods. The plot shows means and 95% confidence intervals by sex and brood size treatment.

Growth trajectories of body mass, wing length and sternum length for nestlings that survived up to fledgling differed between treatments (Table S2; Figure 1). Overall, nestlings from enlarged broods grew slower than those in reduced broods, especially in the 20–40 days after hatching (Figure S1), but then they were able to partially catch up in size in the last 10–15 days of development, notably by delaying fledging by 3.16 days (SE = 0.79, *p* < 0.001; Table S6; Figure 2b) compared to nestlings in reduced broods. Accordingly, on day 30, nestlings in enlarged broods were on average 9.6% lighter (EMMs contrast [enlarged − reduced] ± SE = −9.42 g ± 2.32, *p* < 0.001), their wings were 7.2% shorter (−9.88 mm ± 1.88, *p* ≤ 0.001) and their sternum 7.6% shorter (−2.73 mm ± 0.42, *p* < 0.001), when compared to nestlings in reduced broods. Those differences between treatments declined, on day 50, to 5.3% for body mass (EMMs contrast [enlarged − reduced] ± SE = −4.95 g ± 1.66, *p* = 0.003), to 3.5% for wing length (−7.58 mm ± 2.30, *p* = 0.001) and to 2.5% for sternum length (−0.99 mm ± 0.27, *p* < 0.001). Despite delaying fledging, and in turn growing for a longer period, fledglings in enlarged broods remained lighter by 5.2% for body mass (EMMs contrast [enlarged − reduced] ± SE = −4.78 g ± 1.46, *p* = 0.001), shorter by 0.9% for wing length (−2.08 mm ± 0.91, *p* = 0.023) and by 1.6% for sternum length (−0.64 mm ± 0.24, *p* = 0.007) when compared to fledglings in reduced broods at the time of fledging.

In addition to differences in average trait values, brood enlargement increased phenotypic variability early in development (Table 1). On day 30, residual variability in nestlings from enlarged broods was 53% higher for body mass, 40% higher for wing length and 86% higher for sternum length compared to nestlings from reduced broods. Higher variability in enlarged broods persisted at day 50 for body mass (43%) and wing length (169%), whereas differences in sternum length variability were smaller and not statistically supported (Table 1). By the time of fledging, residual variability was similar between the two treatments (Table 1).

**TABLE 1:** Effects of brood size manipulation treatment on residual variability in body mass (g), wing length (mm) and sternum length (mm) at day 30, day 50, and fledging.

	Body mass (g)		Wing length (mm)		Sternum length (mm)	
	SD ratio [95% CI]	*p*	SD ratio [95% CI]	*p*	SD ratio [95% CI]	*p*
Day 30	1.53 [1.06, 2.19]	**0.022**	1.40 [1.01, 1.93]	**0.042**	1.86 [1.30, 2.65]	**<0.001**
Day 50	1.43 [1.00, 2.05]	**0.049**	2.69 [1.83, 3.96]	**<0.001**	1.36 [0.98, 1.89]	0.068
Fledging age	0.92 [0.69, 1.21]	0.543	1.37 [0.95, 1.97]	0.091	0.98 [0.72, 1.35]	0.924

*Note*: Residual variation was modelled using the dispersion component of Gaussian mixed models. Values represent SD ratios (enlarged/reduced) with 95% confidence intervals and associated Wald *p*‐values. SD ratios >1 indicate greater residual variability in enlarged broods, whereas values <1 indicate greater variability in reduced broods. Significant results are shown in bold.

Male and female nestlings show similar growth trajectories in both treatments for body mass, wing and sternum length (no significant interaction between treatment and sex) and fledged at similar age in both treatments (sex by treatment interaction: LRT = 1.67, *p* = 0.196). At fledging, males were nevertheless significantly bigger in structural body size than females (wing: EMMs contrast [male − female] ± SE = 3.18 mm ± 0.71, *p* < 0.001; sternum: 0.73 mm ± 0.19, *p* < 0.001), but both sexes fledged at a similar age (−0.32 days ± 0.52, *p* = 0.536) and with similar body mass (1.27 g ± 1.27, *p* = 0.318).

Of the 223 nestlings involved in this brood manipulation experiment, 169 survived to fledging (75.8%). Survival probability differed between treatments, but not between sexes nor between different years (see Table S4). Nestlings in enlarged broods had significantly lower odds of surviving to fledging than those in reduced broods (OR = 0.32, *p* = 0.025). Model‐based predicted survival probabilities, averaged over years and sexes, were 0.91 (95% CI: 0.80–0.97) in reduced broods and 0.78 (95% CI: 0.66–0.86) in enlarged broods. Predicted survival probabilities for males and females were 0.83 (95% CI: 0.71–0.91) and 0.88 (95% CI: 0.77–0.94), respectively. Time‐to‐event analysis yielded consistent results, as nestlings in reduced broods experienced 59% lower mortality risk than those in enlarged broods (hazard ratio = 0.41, 95% CI: 0.18–0.93, *p* = 0.034). Sex and year did not significantly affect mortality risk (Table S5).

### 3.2 Recruitment from fledging to adulthood

Of all the hatchlings involved at the start of this brood size manipulation experiment, recruitment probability was significantly higher for those in reduced broods compared to enlarged broods (LRT = 4.67, *p* = 0.031; see Table S7). Males and females show similar recruitment rates (LRT = 0.25, *p* = 0.619), irrespective of natal treatment (i.e. non‐significant interaction between treatment and sex: LRT = 0.18, *p* = 0.675). Recruitment varied among years (LRT = 10.15, *p* = 0.006), with lower probabilities in 2004 and 2006 relative to 2003. Averaged across years and sexes, recruitment probability was approximately 28.3% in reduced broods and 11.9% in enlarged broods (OR = 2.93, *p* = 0.027). Among fledged nestlings (*n* = 169), 51 individuals were recruited (30.2%). When analysing the recruitment conditional on fledging, the treatment effect became almost significant, with fledglings from reduced broods having 2.4 times higher odds of recruitment than fledglings from enlarged broods (OR = 2.44; *p* = 0.055). When testing the effect of fledging age on the recruitment probability into adulthood, we found a significant quadratic relationship (quadratic term: LRT = 9.64, *p* = 0.002), with recruitment probability increasing to a peak at intermediate fledging ages and declining sharply at older ages (see Table S8; Figure S2).

**FIGURE 3:**
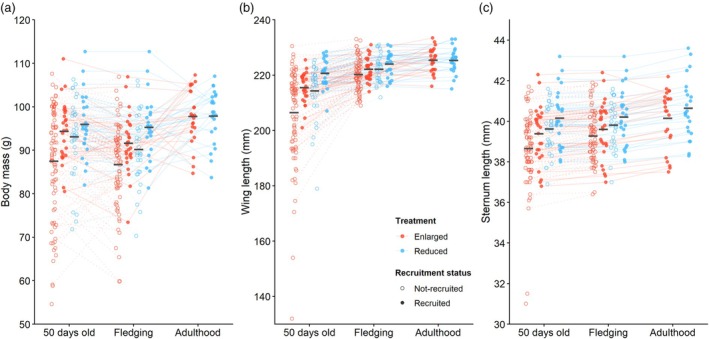
Morphological traits in relation to brood size manipulation treatment and adult recruitment status across life stages. Body mass (a), wing length (b) and sternum length (c) measured at three developmental stages (50 days old, fledging and adulthood). Repeated measurements from the same individual are connected by lines. Points and lines are coloured by brood size treatments (enlarged: Red; reduced: Blue). Recruited individuals are shown with filled points and solid lines, whereas non‐recruited individuals are shown with open points and dashed lines. Horizontal black bars indicate the mean value for each treatment and recruitment group at each stage.

At 50 days of age, treatment differences in size were evident among individuals that were not recruited but weaker and not statistically supported among those that recruited (Figure 3; Table S9). Nestlings from enlarged broods were smaller than those from reduced broods among the non‐recruited subset (EMMs contrast [enlarged − reduced] [95% CI]: mass −5.04 g [−9.01, −1.07]; wing −8.12 mm [−13.60, −2.59]; sternum −0.91 mm [−1.57, −0.24]). Among recruited individuals, the corresponding differences between treatments were smaller and not statistically supported (EMMs contrast [enlarged − reduced] [95% CI]: mass −3.78 g [−8.90, 1.34]; wing −5.68 mm [−12.70, 1.34]; sternum −0.81 mm [−1.70, 0.08]). The size differences measured at fledging showed a similar pattern, with nestlings from enlarged broods tending to be smaller than those from reduced broods among non‐recruited individuals, while differences among recruited individuals were weaker (Table S9). Among individuals that recruited, adult morphology did not differ between birds reared in enlarged and reduced broods (all *p* > 0.25; Table S10). Adult body mass varied with time of capture during the day and across the breeding season, while wing length increased slightly with age, but none of the morphological traits showed evidence of a persistent treatment effect in adulthood (Table S10). Adult morphology was correlated with fledging size within individuals, with positive associations for wing length (Pearson correlation: *r* = 0.56, *p* < 0.001, *n* = 47) and sternum length (*r* = 0.98, *p* < 0.001, *n* = 47), but not for mass (*r* = 0.14, *p* = 0.337, *n* = 47; Figure S3).

**FIGURE 4:**
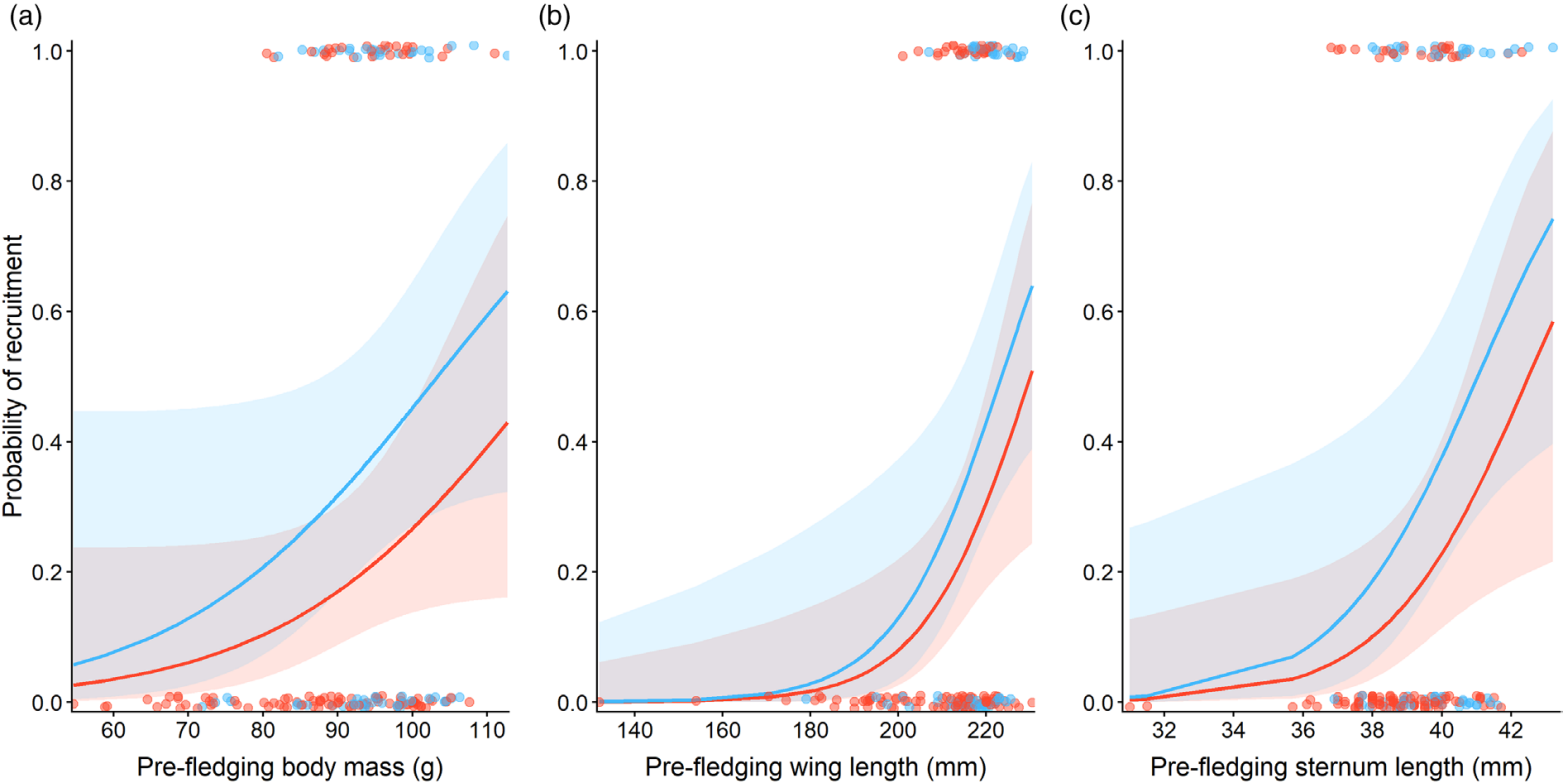
Predicted probability of recruitment into adulthood as a function of morphological traits measured at day 50 (pre‐fledging): Body mass (a), wing length (b) and sternum length (c). Points show observed recruitment outcomes (0 = not recruited, 1 = recruited). Solid lines represent model‐predicted probabilities for each brood size treatment (reduced: Blue; enlarged: Red), and shaded areas indicate the 95% confidence intervals.

Recruitment probability into adulthood increased with body size pre‐fledging (i.e. size measured at day 50 after hatching; Figure 4). The interaction between size and treatment was not significant for any trait (mass: LRT = 0.21, *p* = 0.645; wing: LRT = 0.99, *p* = 0.320; sternum: LRT = 0.25, *p* = 0.617), indicating that the relationship between body size and recruitment probability did not differ between treatments. Recruitment probability increased significantly with mass, wing length and sternum length (all *p* < 0.042; Table S11). The odds of recruitment increased by approximately 5.9% per gram of body mass (OR of 1.88 per 1 SD), 8.5% per mm of wing length (OR of 3.11 per 1 SD), and 62.6% per mm of sternum length (OR of 2.23 per 1 SD).

### 3.3 Adult life‐history traits

#### 3.3.1 Timing and duration of the reproductive career

Birds reared in experimentally reduced or enlarged broods and recruited as adults into the population began breeding at the age of 2–5 years (mean ± SD: 2.90 ± 0.79), and lived up to the age of 2–15 years (mean ± SD of 6.09 ± 3.46), which is within the age ranges reported for Alpine swifts (Tettamanti et al., 2012). There were no significant differences in age at first reproduction between natal treatments (LRT = 0.04, *p* = 0.836), between sexes (LRT = 2.14, *p* = 0.143) or in the interaction between sex and treatment (LRT = 0.00, *p* = 0.960; Table S12; Figure S4). Similarly, there were no significant differences in lifespan between natal treatments (LRT = 0.77, *p* = 0.381), between sexes (LRT = 1.40, *p* = 0.237), or in the interaction between sex and treatment (LRT = 2.44, *p* = 0.119). Reproductive lifespan ranged from 1 to 13 years (mean ± SD: 3.79 ± 3.20), and did not differ between natal treatments (LRT = 1.32, *p* = 0.250), sexes (LRT = 0.01, *p* = 0.921), or their interaction (LRT = 1.80, *p* = 0.179). However, when analysing the number of breeding attempts during the reproductive career, results show a significant interaction between treatment and sex (LRT = 4.96, *p* = 0.026; see Table S13). Females from enlarged broods attempted to breed 2.25 times more than those from reduced broods (EMMs ± SE: 4.24 ± 0.82 vs. 1.89 ± 0.52; rate ratio = 2.25, *p* = 0.017), whereas no treatment difference was detected among males (rate ratio = 0.73, *p* = 0.385). This effect on the number of breeding attempts was partly explained by the tendency of females from enlarged broods to have a longer reproductive lifespan (rate ratio = 1.85, *p* = 0.083; Table S15) as well as to be observed breeding more frequently during those years (OR = 2.79, *p* = 0.096; Table S14). Dividing the number of breeding attempts by reproductive lifespan showed that females from enlarged broods were observed breeding in 91% of the years of their breeding lifespan, compared with 84% for females from reduced broods.

#### 3.3.2 Lifetime reproductive success

**FIGURE 5:**
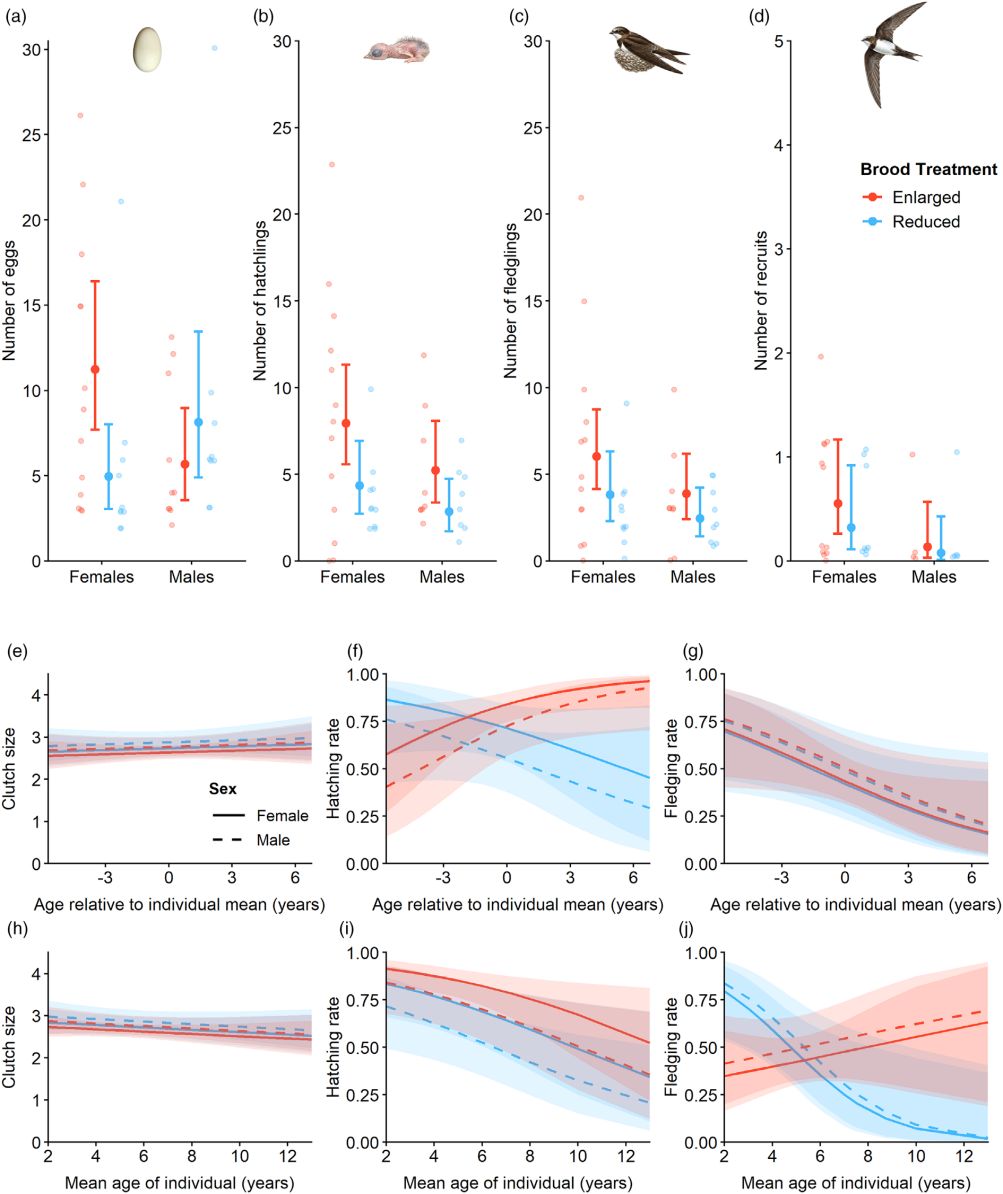
Effects of brood size manipulation on lifetime and age‐specific reproductive performance. (a–d) Lifetime reproductive success components for individuals reared in enlarged (red) and reduced (blue) broods, shown separately for females and males: Total number of eggs (a), total number of hatchlings (b), total number of fledglings (c) and total number of recruits (d). Points represent individual values and error bars indicate model‐estimated means ± 95% confidence intervals. (e–g) Within‐individual effects on annual reproductive output, where age is expressed relative to each individual's mean age. (h–j) Between‐individual effects on annual reproductive output, where age represents the individual's mean age across observations. Responses are annual clutch size (e, h), hatching rate (f, i) and fledgling rate (g, j). Lines represent model predictions for females (solid) and males (dashed), with shaded areas indicating the 95% confidence intervals. Number of individuals (*N*) and observations (*n*) for males in enlarged broods (*N* = 10, *n* = 23), males in reduced broods (*N* = 9, *n* = 28), females in enlarged broods (*N* = 13, *n* = 54), females in reduced broods (*N* = 10, *n* = 22).

Lifetime reproductive success differed between natal treatments for several reproductive measures (Figure 5a–d; Table S16). Total number of eggs produced showed a significant treatment by sex interaction (LRT = 6.13, *p* = 0.013). Females reared in enlarged broods produced 2.27 times more eggs over their lifetime than females from reduced broods (rate ratio = 2.27, *p* = 0.010), whereas no treatment difference was detected among males (rate ratio = 0.70, *p* = 0.282). Lifetime number of hatchlings was significantly higher in birds reared in enlarged broods compared with reduced broods (LRT = 5.03, *p* = 0.025), with no evidence of a treatment by sex interaction (LRT = 1.60, *p* = 0.206). On average, birds reared in enlarged broods produced about 1.83 times more hatchlings over their lifetime than those from reduced broods (rate ratio = 1.83, *p* = 0.020). The number of fledglings showed a similar trend but the treatment effect was not statistically significant (LRT = 2.44, *p* = 0.119). The number of recruited offspring did not differ between natal treatments (LRT = 0.76, *p* = 0.383). When lifetime reproductive output was analysed while accounting for the number of breeding attempts, there was no evidence that birds from enlarged broods produced more eggs per attempt (LRT = 0.48, *p* = 0.489), and only weak evidence for higher hatchling production per attempt (LRT = 2.96, *p* = 0.085; see Table S17).

#### 3.3.3 Age‐specific reproductive performance

Age‐specific reproductive performance showed distinct within‐ and between‐individual patterns across different reproductive measures (Figure 5e–j; Table S18). Annual clutch size did not vary with age either within or among individuals and showed no differences between natal treatments (treatment by age interactions: all *p* > 0.294). Hatching success showed treatment‐specific within‐individual age trajectories (treatment by age within: LRT = 6.06, *p* = 0.014). Individuals from enlarged broods showed an increase in hatching success with age within individuals (slope [95% CI]: 0.24 [0.01, 0.46] per year), whereas those from reduced broods tended to decline (slope [95% CI]: −0.16 [−0.40, 0.07] per year). Between individuals, hatching success declined with mean age in both treatments (slope [95% CI]: −0.21 [−0.37, −0.04] per year), with birds from enlarged broods tending to have higher hatching success across the age gradient (Figure 5i). Fledging success declined with age within individuals similarly across treatments (treatment by age within: LRT = 0.04, *p* = 0.843; Figure 5g). In contrast, between individuals, fledging success tended to increase slightly with mean age in birds from enlarged broods (slope [95% CI]: 0.11 [−0.15, 0.36] per year), whereas it declined in birds from reduced broods (slope [95% CI]: −0.49 [−0.89, −0.10] per year; Figure 5j). Results for the number of hatchlings and number of fledglings are presented in the Supporting Information (Figure S5; Table S18).

#### 3.3.4 Environmental quality and reproductive performance

The response of reproductive success to variation in annual environmental conditions did not differ between individuals reared in enlarged and reduced broods (treatment by year‐quality interactions: all *p* > 0.142; see Table S19; Figure S6). After removing the interaction term from the model, environmental quality index was positively associated with hatching rate, fledging rate and the number of fledglings produced (year‐quality all *p* < 0.015), negatively associated with clutch size (LRT = 4.45, *p* = 0.035), and not associated with the number of hatchlings (LRT = 2.26, *p* = 0.133).

## 4 DISCUSSION

Early‐life environmental conditions are expected to influence individual survival and reproductive success. Many studies have demonstrated that adverse conditions and resource limitation during development can constrain growth and impose long‐lasting fitness costs (Cooper & Kruuk, 2018; Reid et al., 2003; van de Pol et al., 2006), while others reported the absence of such effects (Cam et al., 2003; Drummond & Rodríguez, 2013; Vedder et al., 2022; Vedder & Bouwhuis, 2018). To test how variation in developmental conditions shapes life‐history trajectories, we experimentally manipulated sibling competition through brood size enlargement and reduction in a wild population of a long‐lived bird species. The experiment involved 90 Alpine swift nests with 223 nestlings that were monitored throughout their life. This approach allowed us to study both the immediate developmental consequences and the lifetime fitness outcomes for these individuals. We found that brood enlargement reduced the average growth and size and increased phenotypic variability among nestlings of enlarged broods. These developmental differences translated into viability selection. Nestlings reared in enlarged broods had lower survival to fledging and lower recruitment probability than those reared in reduced broods. Across both treatments, recruitment probability increased with pre‐fledging size. As a result, morphological differences between treatments largely disappeared among individuals that survived to adulthood. Additionally, the recruits from enlarged broods performed well as adults and in some cases showed higher reproductive performance than those reared in reduced broods. Here, we discuss these findings in the context of sibling competition during development, viability selection and selective disappearance, and the implications of missing‐fraction problems for interpreting the fitness consequences of adverse early‐life environment.

### 4.1 Early‐life competition increases developmental variability

We found that nestlings from enlarged broods were smaller in size and lighter compared to nestlings reared in reduced broods (Figure 1; Table S3). This result is consistent with numerous observational and experimental studies showing that offspring reared in larger broods often experience reduced growth (e.g. Burness et al., 2000; Cucco & Malacarne, 1996; Dijkstra et al., 1990; Kogel, 1997; Sicurella et al., 2015; Tschirren et al., 2009; Vitousek et al., 2017). As brood size increases, parental provisioning per capita typically declines, resulting in stronger competition among nestlings for limited food resources (Siefferman & Hill, 2007). Although we did not measure feeding rates directly, a reduction in food provisioning per capita with increasing brood size was reported in two other swift species with a very similar ecology (pallid swift *Apus pallidus*: Cucco & Malacarne, 1996; common swift *Apus apus*: Martins, 1993).

Differences in body size between treatments were most pronounced at day 30 after hatching and persisted until fledging despite evidence of partial catch‐up growth (Figure 1; Figure S1). Such compensatory responses may involve accelerated growth when food becomes abundant or extend the developmental window and delay the maturation of specific tissues when resources are still scarce (Bize et al., 2003; Metcalfe & Monaghan, 2001; Schew & Ricklefs, 1998). In our study, compensation differed among tissues: differences in wing length and sternum length diminished more strongly than those in body mass (see Section 3.1). Differential compensation among traits is expected because tissues vary in their functional importance and energetic costs, with traits closely related to survival and locomotion often being prioritized during development (Boonekamp et al., 2018; Mangel & Munch, 2005; Stearns & Kawecki, 1994). The stronger compensation observed in wing length may reflect the close link between flight performance and survival, leading this trait to be more tightly canalized during development. Although a previous study showed that Alpine swift nestlings were more likely to prioritize rapid short‐term catch‐up in body mass (i.e. body reserves) over sternum and wing length (Bize et al., 2005), this was in response to a short and strong food shortage caused by poor weather conditions rather than a long but moderate reduction in food caused by increasing brood size throughout development. In our study, nestlings from enlarged broods fledged about 3 days later than those from reduced broods, suggesting that they prolonged the nestling period to compensate for earlier growth deficits. Nevertheless, compensation was incomplete and individuals from enlarged broods remained smaller at fledging. This partial catch‐up likely occurs because swift parents reduce feeding their nestlings close to fledging, thus driving them to fledge from hunger and preventing them from remaining much longer in the nest.

Importantly, we found that brood enlargement increased phenotypic variability among nestlings (Table 1). While some nestlings were strongly affected by increased competition, others developed similarly to nestlings from reduced broods. Such heterogeneous responses are expected when resources are limited because unequal access to food can amplify differences in competitive ability or intrinsic quality among individuals (Łomnicki, 1978). Therefore, the variance in individual quality should increase for cohorts born under poor environmental conditions (Hamel et al., 2009; McNamara, 1998).

In species with parental care, the developmental environment can also be shaped by parental allocation decisions, which may buffer or exacerbate such variability in offspring quality (Bize, Piault, et al., 2006; Descamps et al., 2016). In some systems, parents may even promote competitive asymmetries among their offspring, because stable hierarchies and inducing rapid mortality of low‐ranked offspring can reduce the energetic costs of sibling competition for parents (Hahn, 1981; Vedder et al., 2017, 2019). In our study system, eggs within a brood hatch synchronously and the experimental design ensured that cross‐fostered nestlings were similar in size and age to their resident siblings and to their new nest‐mates (Bize et al., 2010; Bize, Gasparini, et al., 2006; Bize & Roulin, 2006; Roulin et al., 2008), reducing the potential for strong size‐based hierarchies at the start of development. Instead, increased brood size likely generated heterogeneity in development through subtle differences in offspring competitive ability and resource acquisition, as well as parental favoritism and food allocation during growth. By increasing phenotypic variance among nestlings, brood enlargement effectively amplified the variation in individual quality on which natural selection can act.

### 4.2 Viability selection filters individuals before recruitment

Nestling survival from hatching to fledging was 15% lower in enlarged broods compared to reduced broods (Figure 2), a pattern consistent with previous brood size manipulation studies (Dijkstra et al., 1990; Kogel, 1997). This reduced survival likely reflects increased sibling competition, with individuals of lower competitive ability failing to secure sufficient resources and dying before fledging. As a consequence, overall recruitment probability across all hatchlings was lower in enlarged broods and largely driven by lower pre‐fledging survival. However, even among fledged individuals, those from reduced broods were 2.4 times more likely to recruit than those from enlarged broods.

We found no evidence of sex‐specific effects of brood size manipulation on nestling survival, recruitment, or lifespan (Tables S4, S7, and S12). However, recruitment probability was strongly associated with pre‐fledging size (Figure 4; Table S11). Larger nestlings were more likely to recruit, with wing length showing the strongest association with recruitment probability. This pattern indicates that viability selection acted on phenotypic variation generated during development, with larger nestlings being more likely to recruit into adulthood. The timing of fledging further supports this interpretation. Individuals fledging at intermediate ages (around day 55) had the highest probability of recruitment, whereas those fledging later (predominantly from enlarged broods) showed reduced recruitment (Figure S2). Although delayed fledging may allow additional time for growth, these individuals developed under prolonged constraints and remained of lower quality. Compensatory growth under such conditions is known to carry costs, which can reduce survival and fitness even if short‐term growth deficits were partially compensated (Metcalfe & Monaghan, 2001, 2003).

Morphological differences between treatments were pronounced among nestlings that did not recruit, but decreased among individuals that later recruited (Figure 3; Table S9). Accordingly, there was no difference in morphology between treatments at adulthood (Tables S9 and S10). Because Alpine swifts exhibit determinate growth and cease growing after fledging (Masoero et al., 2024), as demonstrated by the moderate to strong correlation in wing and sternum length at fledging and adulthood within the same individuals (Figure S3), the disappearance of treatment differences in adulthood is unlikely to be attributed to post‐fledging growth. Instead, it reflects selective mortality that removed smaller individuals from the adult population. This pattern is consistent with a ‘missing fraction’ problem, whereby individuals most affected by early‐life conditions are selectively removed before adulthood, biasing the phenotypic composition of the surviving cohort (Grafen, 1988).

Viability selection could be particularly strong in this species due to the abrupt transition from nest life to continuous flight. Alpine swifts receive no post‐fledging parental care (Arn, 1960; Lack, 1956; PB, pers. obs.), and juveniles must immediately sustain prolonged flight and forage independently after fledging (Hedenström, 2010; Liechti et al., 2013). Under these conditions, achieving sufficient body size and flight capacity at fledging is crucial for survival. Consistent with this, we found that selection favoured larger individuals (Figure 4), especially those with longer wings, a trait closely linked to flight performance (Martin et al., 2018). Therefore, adults originating from enlarged broods represent a non‐random subset of individuals that were relatively less affected by early‐life competition. Further support for the occurrence of viability selection in our system comes from Moullec et al. (2025), who studied the lifelong effects of parental age at conception on offspring phenotype. These authors found similar positive lifelong effects of a harsh early environment—measured as annual nestling mortality—on offspring life‐history trajectories.

### 4.3 Adult reproductive performance

Although there were no differences between natal treatments in age at first reproduction (Figure S4; Table S12), females originating from enlarged broods tended to have longer lifespan and reproductive career, resulting in almost twice as many breeding attempts over their lifetime than females from reduced broods (Table S15); no such difference was detected among males. Moreover, recruits from enlarged broods showed equal or higher lifetime reproductive output (Figure 5; Table S16). In particular, the total number of hatchlings produced over their lifetime was higher in individuals reared in enlarged broods, with a similar but non‐significant trend for the number of fledglings. Importantly, these differences were not explained by higher reproductive output per season when accounting for the number of breeding attempts (Table S17).

These results might suggest that individuals exposed to harsher early‐life conditions perform better as adults. However, such interpretation would be misleading. Brood enlargement reduced recruitment probability, indicating that overall fitness across the full life cycle was lower for this treatment. When individuals that failed to recruit are considered, there is no evidence that early‐life stress increased fitness. As shown in Section 4.2, individuals most strongly affected by increased sibling competition were less likely to survive to adulthood. Therefore, the high reproductive performance observed among these individuals likely reflects selective disappearance of lower quality individuals.

Sex‐specific effects on reproductive success were generally weak. The higher lifetime egg production observed in females from enlarged broods represents the only reproductive trait showing a treatment difference within one sex, whereas most other reproductive measures, including hatchling and fledgling production and age‐specific reproductive performance, were similar between sexes among treatments (Tables S16 and S18). Females also produced more recruiting offspring than males, but recruitment numbers were low and this pattern should be interpreted cautiously, as stochastic variation can strongly influence such estimates. Overall, our results provide little evidence for strong sex‐specific effects of early‐life conditions on adult reproductive trajectories.

### 4.4 Age‐specific reproductive patterns and selective disappearance

By decomposing age into within‐ and between‐individual components, we show that age‐specific reproductive patterns emerged from the combined effects of within‐individual senescence and selective disappearance among breeding adults (Table S18). Clutch size remained stable both within and among individuals, suggesting that this trait does not exhibit detectable senescence or selective disappearance (Moullec et al., 2023). This is consistent with studies in long‐lived species, where clutch size often shows weak or delayed senescence (Bouwhuis et al., 2009; Hammers et al., 2012; McCleery et al., 2008).

In contrast, hatching success varied with age (within‐ and between‐individual effects) in interaction with treatment. Within individuals, hatching success increased with age in birds from enlarged broods, consistent with increasing experience and selection for higher‐quality individuals, whereas individuals from reduced broods tended to decline, indicating a trend towards lower quality individuals more prone to senescence. For fledging success, within‐individual effects showed a decline with age in both treatments (i.e. senescence). However, between‐individual patterns diverged: individuals from enlarged broods tended to slightly increase fledging success with age, whereas those from reduced broods declined. This suggests that individuals from enlarged broods that survive to older ages represent a higher‐quality subset and maintain higher reproductive performance at older ages than those from reduced broods (i.e. selective disappearance of lower quality breeders).

Evidence for long‐term effects of early‐life conditions on reproductive senescence is mixed. Some studies report no detectable effect of developmental conditions on age‐specific reproduction or survival (Vedder et al., 2022; Vedder & Beccardi, 2025), whereas others found that poor early‐life conditions accelerate senescence or reduce late‐life performance (Nussey et al., 2007), or that favorable conditions are associated with slower rates of senescence (Cooper & Kruuk, 2018).

In our study system, previous work has shown that reproductive performances typically increase during early‐life, reflecting the accumulation of experience (Forslund & Pärt, 1995; Saraux & Chiaradia, 2022), before declining at older ages, particularly in females (Moullec et al., 2023). The mechanisms underlying reproductive senescence may involve deterioration in incubation behaviour, egg quality or fertilization success (Bouwhuis et al., 2009; Lemaître & Gaillard, 2017). Notably, senescence effects become more pronounced across successive stages of reproduction, from no detectable change in clutch size to clear declines in brood size at hatching and fledging (Moullec et al., 2023). This pattern suggests that reproductive senescence results from the cumulative costs of reproduction across stages, from egg production to incubation and nestling provisioning. In addition, hatching and fledging success also depend on offspring quality, not only on parents' performance. It is, however, important to notice that all the present results on reproductive patterns are based on small sample sizes; they should therefore be interpreted with caution.

### 4.5 No evidence for match–mismatch effects on adult reproductive success

We found no evidence that early‐life conditions interacted with environmental conditions experienced during adulthood (Figure S6; Table S19). Although annual environmental quality influenced reproductive success, the response to environmental variation was similar for individuals from enlarged and reduced broods. This contrasts with predictions from a match–mismatch (or predictive adaptive response) framework, which predicts that individuals should perform best when adult conditions resemble those experienced during development (Monaghan, 2007). Our results indicate that early‐life conditions did not alter how individuals responded to environmental variation later in life.

## 5 CONCLUSIONS

Our results show that early‐life conditions can generate substantial developmental heterogeneity, which interacts with strong viability selection to shape adult phenotypes. Increased sibling competition amplified variation among individuals, leading to the preferential survival of those least affected by early‐life constraints. More broadly, such processes resemble strategies observed in some long‐lived species, where early mortality can reduce variation in offspring quality at independence and contribute to more homogeneous adult cohorts in stochastic environments (Vedder & Bouwhuis, 2018).

This combination can produce counterintuitive patterns, where individuals from poor developmental environments appear to perform as well as, or better than, those from more favorable conditions. Such patterns reflect selective (dis)appearance rather than beneficial effects of early‐life stress, which otherwise might be misinterpreted as hormesis (Calabrese & Baldwin, 2002; Costantini, 2010, 2019; Forbes, 2000). Accordingly, selective disappearance and missing‐fraction problems can bias inferences about long‐term fitness when analyses are restricted to individuals that survive to adulthood, as survivors are unlikely to represent a random sample of their cohort (Garratt et al., 2015; Grafen, 1988; Vaupel et al., 1979).

These findings refine the interpretation of silver‐spoon effects, which may conflate causal effects of developmental conditions with selective survival. By experimentally manipulating early‐life conditions and following individuals across their lifespan, we show that early adversity can reduce overall fitness while still producing a subset of high‐performing survivors. Finally, we found no support for predictive adaptive response or match–mismatch effect (Gluckman & Hanson, 2004; Monaghan, 2007), indicating that early‐life conditions primarily influenced survival to adulthood, with no evidence for effects on adult plasticity.

## AUTHOR CONTRIBUTIONS

Pierre Bize and Giulia Masoero conceived the study, Pierre Bize designed methodology and collected the data; Bashar Jarayseh led the analyses of the data with support from Giulia Masoero and Pierre Bize; Bashar Jarayseh and Pierre Bize led the writing of the manuscript. All authors contributed critically to the drafts and gave final approval for publication.

## CONFLICT OF INTEREST STATEMENT

The authors have no conflicts of interest to declare.

## STATEMENT ON INCLUSION

Our study brings together authors from different countries, including scientists based in the country where the study was carried out. All authors were engaged early on with the data analysis strategy to ensure that the diverse sets of perspectives they represent were considered from the onset; however, the analyses are based on long‐term experimental data, which precluded the early inclusion of all the authors in the data collection. Whenever relevant, literature published by scientists from the region was cited; efforts were made to consider relevant work published in the local language.

## Supporting information


**Table S1.** Number of nests per treatment group across the study years, with the reproductive outcomes shown as the total number of nestlings, fledglings, and individuals recruited into the population, categorized by year, treatment, and sex.
**Table S2.** Results of generalized additive models (Gaussian error distribution) testing the effects of brood size manipulation treatment, sex, and hatching year on growth trajectories of mass (g), wing length (mm), and sternum length (mm) in Alpine swift nestlings.
**Table S3.** Results of linear mixed‐effects models testing the effects of brood‐size manipulation treatment, sex, and hatching year on morphological traits measured during development (day 30, day 50, and fledging).
**Table S4.** Results of a generalized linear mixed‐effects model testing the effects of brood‐size manipulation treatment, sex, and hatching year on fledging success.
**Table S5.** Results of a Cox proportional hazards model testing the effects of brood‐size manipulation treatment, sex, and hatching year on survival.
**Table S6.** Results of a linear mixed‐effects model testing the effects of brood‐size manipulation treatment, sex, and hatching year on fledging age in days.
**Table S7.** Results of generalized linear mixed‐effects models testing the effects of brood‐size manipulation treatment, sex, and hatching year on recruitment probability to adulthood.
**Table S8.** Results of a generalized linear mixed‐effects model testing the relationship between fledging age (in days) and recruitment probability to adulthood.
**Table S9.** Estimated marginal means of morphological traits measured at three developmental stages (50 days of age, fledging age, and adulthood) from Gaussian mixed‐effects models.
**Table S10.** Results of linear mixed models testing the effects of brood size manipulation treatment, sex, and hatching year on the morphometric traits of adult birds reared in these broods.
**Table S11.** Results of generalized linear mixed‐effects models testing the association between morphological traits measured on day 50 after hatching (body mass, wing length, and sternum length) and recruitment probability.
**Table S12.** Results of linear models testing the effects of brood‐size manipulation treatment, sex, and hatching year on three life‐history traits: age at first reproduction, lifespan, and reproductive lifespan.
**Table S13.** Results of a generalized linear model testing the effects of brood‐size manipulation treatment, sex, hatching year, and the interaction between treatment and sex on the number of breeding attempts during an individual's reproductive lifetime.
**Table S14.** Results of a generalized linear model testing the effects of brood‐size manipulation treatment, sex, and hatching year on the annual breeding probability.
**Table S15.** Estimated marginal means for age at first reproduction, lifespan, reproductive lifespan, and number of breeding attempts (breeding years).
**Table S16.** Results of generalized linear models testing the effects of brood size manipulation treatment, sex, and hatching year on lifetime reproductive success, measured as the total number of eggs, hatchlings, fledglings, and recruits produced by each individual.
**Table S17.** Results of generalized linear models testing the effects of brood‐size manipulation treatment, sex, and hatching year on annual reproductive success, measured as the number of eggs and hatchlings produced per breeding attempt.
**Table S18.** Results of mixed‐effects models testing the effects of brood‐size manipulation treatment, sex, hatching year, and age on age‐specific reproductive performance.
**Table S19.** Results of mixed‐effects models testing the effects of brood‐size manipulation treatment, environmental quality, sex, age, and hatching year on annual reproductive performance, measured as clutch size, number of hatchlings, hatching rate, number of fledglings, and fledging rate per breeding attempt.
**Figure S1.** Model predicted difference in body mass (g; A), wing length (mm; B), and sternum length (mm; C), with 95% CI, between Alpine swift nestlings reared in experimentally enlarged and reduced broods, in relation to age.
**Figure S2.** Predicted probability of recruitment of Alpine swift nestlings as a function of fledging age.
**Figure S3.** Relationship between size measured at fledging and during adulthood for (A) mass, (B) wing length, and (C) sternum length of Alpine swifts reared in experimentally enlarged (red) and reduced (blue) broods.
**Figure S4.** Variation in age at first reproduction (A), lifespan (B), and reproductive lifespan (C) of Alpine swifts reared in experimentally enlarged (red) and reduced (blue) broods and recruited as adults in the population.
**Figure S5.** Within‐ and between‐individual effects of age on annual reproductive output.
**Figure S6.** Relationship between annual environmental quality and reproductive performance.

## Data Availability

All code and data to reproduce the analyses and figures in this manuscript are available on the Open Science Framework repository https://doi.org/10.17605/OSF.IO/4RFCX (Jarayseh et al., 2026).
