# Supplementary material for: The role of viability selection in shaping short‐ and long‐term consequences of early‐life conditions: An experiment in the Alpine swift
Source: J Anim Ecol. 2026 Jul 13;95(8):1401–18. doi: 10.1111/1365-2656.70314 (PMC13439603; doi:10.1111/1365-2656.70314)
Supplement: Supplementary file 1 [file JANE-95-1401-s001.docx]

**Supporting Information for**

**The role of viability selection in shaping short- and long-term consequences of early-life conditions: An experiment in the Alpine swift**

**Bashar Jarayseh, Giulia Masoero, Pierre Bize**

**Table S1.** Number of nests per treatment group across the study years, with the reproductive outcomes shown as the total number of nestlings, fledglings, and individuals recruited into the population, categorized by year, treatment, and sex. M:F represents the number of males to the number of females.

| **Treatment** | **Year** | **Nests** | **Nestlings** | **Fledglings (M:F:NA)** | **Recruits (M:F)** |
| --- | --- | --- | --- | --- | --- |
| **Reduced** | 2003 | 16 | 27 | 24 (12:12) | 17 (9:8) |
|  | 2004 | 14 | 19 | 16 (9:7) | 3 (2:1) |
|  | 2006 | 15 | 22 | 19 (8:11) | 4 (1:3) |
|  | sum | 45 | 68 | 59 (29:30) | 24 (12:12) |
| **Enlarged** | 2003 | 16 | 56 | 47 (19:28) | 13 (6:7) |
|  | 2004 | 14 | 49 | 31 (14:16:1) | 9 (2:7) |
|  | 2006 | 15 | 50 | 32 (19:13) | 5 (3:2) |
|  | sum | 45 | 155 | 110 (52:57:1) | 27 (11:16) |
| **Total** |  | 90 | 223 | 169 (81:87:1) | 51 (23:28) |

**Table S2.** Results of generalised additive models (Gaussian error distribution) testing the effects of brood size manipulation treatment, sex, and hatching year on growth trajectories of mass (g), wing length (mm), and sternum length (mm) in Alpine swift nestlings. Shown are parameter estimates with standard errors for parametric terms and estimated degrees of freedom, F-statistics, and associated p-values for smooth terms. Random effects are reported as standard deviations with 95% confidence intervals. Sample sizes are reported as number of nestlings measured (N) and total number of measurements (n). The effect of treatment is expressed as enlarged vs reduced, sex as male vs. female, and hatching year as 2003 vs 2004 or 2006.

|  | **Body Mass (g)** | | | **Wing Length (mm)** | | | **Sternum Length (mm)** | | |
| --- | --- | --- | --- | --- | --- | --- | --- | --- | --- |
|  | *N* = 173, *n* = 1086 | | | *N* = 173, *n* = 1085 | | | *N* = 173, *n* = 913 | | |
| ***Parametric coefficients*** | ***Estimate ± SE*** | | ***P*** | ***Estimate ± SE*** | | ***P*** | ***Estimate ± SE*** | | ***P*** |
| (Intercept) | 68.70 ± 1.19 | | **< 0.001** | 111.69 ± 1.44 | | **< 0.001** | 30.37 ± 0.28 | | **< 0.001** |
| Treatment (reduced) | 5.56 ± 1.20 | | **< 0.001** | 5.65 ± 1.45 | | **< 0.001** | 1.35 ± 0.28 | | **< 0.001** |
| Sex (female) | -0.83 ± 0.73 | | 0.258 | -1.51 ± 0.95 | | 0.116 | -0.73 ± 0.21 | | **< 0.001** |
| Hatching year (2004) | -1.95 ± 1.47 | | 0.187 | -5.25 ± 1.76 | | **0.004** | -0.80 ± 0.35 | | **0.021** |
| Hatching year (2006) | -4.50 ± 1.43 | | **0.002** | -4.34 ± 1.72 | | **0.013** | -0.13 ± 0.33 | | 0.699 |
| ***Smooth terms*** | ***Edf*** | ***F-value*** | ***P*** | ***Edf*** | ***F-value*** | ***P*** | ***Edf*** | ***F-value*** | ***P*** |
| Age: Treatment (enlarged) | 7.19 | 659.12 | **< 0.001** | 8.58 | 3096.10 | **0.004** | 7.69 | 2386.61 | **0.014** |
| Age: Treatment (reduced) | 6.89 | 448.82 | **< 0.001** | 8.28 | 1797.60 | 0.854 | 6.03 | 1315.50 | 0.132 |
| ***Random effects*** | ***Variance (SD)*** | | ***95% CI*** | ***Variance (SD)*** | | ***95% CI*** | ***Variance (SD)*** | | ***95% CI*** |
| Nest identity | 4.67 | | 3.72 – 5.87 | 5.44 | | 4.30 – 6.89 | 0.98 | | 0.74 – 1.30 |
| Bird identity (linear age effect) | 3.22 | | 2.59 – 4.01 | 5.02 | | 4.32 – 5.83 | 1.09 | | 0.92 – 1.28 |
| Bird identity (nonlinear age deviation) | 1.79 | | 1.49 – 2.14 | 1.87 | | 1.78 – 1.98 | 0.26 | | 0.22 – 0.32 |
| Residual | 4.96 | | 4.37 – 5.63 | 1.08 | | 0.89 – 1.32 | 0.76 | | 0.67 – 0.87 |

**Table S3.** Results of linear mixed-effects models testing the effects of brood-size manipulation treatment, sex, and hatching year on morphological traits measured during development (day 30, day 50, and fledging). Separate models were fitted for body mass, wing length, and sternum length at each developmental stage. Estimates are presented as fixed-effect coefficients ± SE with corresponding P-values obtained from likelihood ratio tests based on single-term deletions from the fitted models. Variance components of the random effect and sample sizes are reported. The effect of treatment is expressed as enlarged vs reduced, sex as female vs. male, and hatching year as 2003 vs 2004 or 2006.

| **Development stage** |  | **Body mass (g)** | | | **Wing length (mm)** | | **Sternum length (mm)** | | |
| --- | --- | --- | --- | --- | --- | --- | --- | --- | --- |
| **Day 30** | **Fixed effects** | ***Estimate ± SE*** | | ***P*** | ***Estimate ± SE*** | ***P*** | ***Estimate ± SE*** | ***P*** | |
|  | (Intercept) | 89.54 ± 2.32 | | - | 128.38 ± 1.87 | - | 33.03 ± 0.43 | - | |
|  | Treatment (reduced) | 9.42 ± 2.32 | | **< 0.001** | 9.88 ± 1.88 | **< 0.001** | 2.73 ± 0.42 | **< 0.001** | |
|  | Sex (male) | 1.02 ± 1.41 | | 0.472 | 1.13 ± 1.34 | 0.397 | 0.59 ± 0.28 | **0.040** | |
|  | Hatching year (2004) | -6.15 ± 2.85 | | **0.025** | -6.18 ± 2.33 | **0.007** | -0.99 ± 0.52 | 0.064 | |
|  | Hatching year (2006) | 1.46 ± 2.77 | | **0.025** | 0.88 ± 2.24 | **0.007** | 0.19 ± 0.51 | 0.064 | |
|  | **Random effects** | **Variance** | | | **Variance** | | **Variance** | | |
|  | Nest identity | 91.22 | | | 49.46 | | 2.78 | | |
|  | Residual | 8.39 | | | 7.69 | | 1.92 | | |
|  | **Sample size** | | | | | | | | |
|  | Nests | 87 | | | 86 | | 87 | | |
|  | Individuals | 187 | | | 186 | | 187 | | |
| **Day 50** | **Fixed effects** | ***Estimate ± SE*** | ***P*** | | ***Estimate ± SE*** | ***P*** | ***Estimate ± SE*** | | ***P*** |
|  | (Intercept) | 93.09 ± 1.67 | - | | 212.60 ± 2.32 | - | 38.61 ± 0.27 | | - |
|  | Treatment (reduced) | 4.65 ± 1.66 | **0.004** | | 7.58 ± 2.30 | **0.001** | 0.99 ± 0.27 | | **< 0.001** |
|  | Sex (male) | 0.40 ± 1.21 | 0.739 | | 2.23 ± 1.28 | 0.093 | 0.75 ± 0.22 | | **0.001** |
|  | Hatching year (2004) | 0.10 ± 3.04 | **< 0.001** | | -5.70 ± 2.83 | **0.001** | -0.16 ± 0.33 | | 0.802 |
|  | Hatching year (2006) | -12.42 ± 2.00 | **< 0.001** | | -10.44 ± 2.76 | **0.001** | -0.20 ± 0.32 | | 0.802 |
|  | **Random effects** | **Variance** | | | **Variance** | | **Variance** | | |
|  | Nest identity | 40.27 | | | 88.22 | | 0.76 | | |
|  | Residual | 6.86 | | | 9.60 | | 1.40 | | |
|  | **Sample size** | | | | | | | | |
|  | Nests | 84 | | | 84 | | 84 | | |
|  | Individuals | 167 | | | 167 | | 166 | | |
| **Fledging age** | **Fixed effects** | ***Estimate ± SE*** | ***P*** | | ***Estimate ± SE*** | ***P*** | ***Estimate ± SE*** | ***P*** | |
|  | (Intercept) | 91.74 ± 1.32 | - | | 220.69 ± 0.88 | - | 39.06 ± 0.22 | - | |
|  | Treatment (reduced) | 4.78 ± 1.46 | **0.001** | | 2.08 ± 0.91 | **0.025** | 0.63 ± 0.23 | **0.008** | |
|  | Sex (male) | -1.27 ± 1.27 | 0.319 | | 3.18 ± 0.71 | **< 0.001** | 0.73 ± 0.19 | **< 0.001** | |
|  | Hatching year (2004) | -1.95 ± 1.68 | **< 0.001** | | -2.02 ± 1.09 | **0.037** | -0.17 ± 0.28 | 0.633 | |
|  | Hatching year (2006) | -9.54 ± 1.71 | **< 0.001** | | -2.77 ± 1.12 | **0.037** | 0.11 ± 0.28 | 0.633 | |
|  | **Random effects** | **Variance** | | | **Variance** | | **Variance** | | |
|  | Nest identity | 10.42 | | | 9.63 | | 0.49 | | |
|  | Residual | 7.03 | | | 3.76 | | 1.04 | | |
|  | **Sample size** | | | | | | | | |
|  | Nests | 80 | | | 80 | | 80 | | |
|  | Individuals | 154 | | | 154 | | 154 | | |

**Table S4.** Results of a generalized linear mixed-effects model testing the effects of brood-size manipulation treatment, sex, and hatching year on fledging success. Fledging success was modelled as a binomial response (fledged vs not fledged). Estimates are presented as fixed-effect coefficients with standard errors and P-values obtained from likelihood ratio tests based on single-term deletions from the fitted model. Variance components of the random effect and sample sizes are reported. The effect of treatment is expressed as enlarged vs reduced, sex as female vs. male, and hatching year as 2003 vs 2004 or 2006.

| **Fixed effects** | ***Estimate*** | ***Std. Error*** | ***P*** |
| --- | --- | --- | --- |
| (Intercept) | 1.89 | 0.47 | - |
| Treatment (reduced) | 1.13 | 0.51 | **0.015** |
| Sex (male) | -0.45 | 0.39 | 0.236 |
| Hatching year (2004) | -0.54 | 0.52 | 0.342 |
| Hatching year (2006) | -0.71 | 0.50 | 0.342 |
| **Random effects** | **Variance** | | |
| Nest identity | 0.57 | | |
| **Sample size** | | | |
| Nests | 89 | | |
| Individuals | 212 | | |

**Table S5.** Results of a Cox proportional hazards model testing the effects of brood-size manipulation treatment, sex, and hatching year on survival. Estimates are presented as hazard ratios (HR) with standard errors and P-values for fixed effects. Nest identity was included as a frailty term to account for non-independence among individuals originating from the same nest. The effect of treatment is expressed as enlarged vs reduced, sex as female vs. male, and hatching year as 2003 vs 2004 or 2006.

| **Predictor** | **HR** | **95% CI** | **z-value** | **P** |
| --- | --- | --- | --- | --- |
| Treatment (reduced) | 0.41 | 0.18 – 0.93 | 4.50 | 0.034 |
| Sex (male) | 1.41 | 0.76 – 2.61 | 1.19 | 0.276 |
| Hatching year (2004) | 1.56 | 0.69 – 3.56 | 1.12 | 0.289 |
| Hatching year (2006) | 1.65 | 0.75 – 3.62 | 1.55 | 0.213 |
| Frailty (Nest identity) | - | - | 8.78 | 0.318 |

**Table S6.** Results of a linear mixed-effects model testing the effects of brood-size manipulation treatment, sex, and hatching year on fledging age in days. Estimates are presented as fixed-effect coefficients with standard errors and P-values obtained from likelihood ratio tests based on single-term deletions from the fitted model. Variance components of the random effect and sample sizes are reported. The effect of treatment is expressed as enlarged vs reduced, sex as female vs. male, and hatching year as 2003 vs 2004 or 2006.

| **Fixed effects** | ***Estimate*** | ***Std. Error*** | ***P*** |
| --- | --- | --- | --- |
| (Intercept) | 55.89 | 0.75 | - |
| Treatment (reduced) | -3.16 | 0.79 | **< 0.001** |
| Sex (male) | 0.32 | 0.52 | 0.537 |
| Hatching year (2004) | 2.89 | 0.94 | **< 0.001** |
| Hatching year (2006) | 4.31 | 0.96 | **< 0.001** |
| **Random effects** | **Variance** | | |
| Nest identity | 8.65 | | |
| Residual | 2.65 | | |
| **Sample size** | | | |
| Nests | 82 | | |
| Individuals | 161 | | |

**Table S7.** Results of generalized linear mixed-effects models testing the effects of brood-size manipulation treatment, sex, and hatching year on recruitment probability to adulthood. Two models are presented: overall recruitment probability (i.e. from hatching to adulthood) and recruitment conditional on fledging (i.e. from fledging to adulthood). Estimates are shown on the logit scale with their standard errors, P-values correspond to likelihood ratio tests based on single-term deletions from the fitted models. Variance components of the random effects and sample sizes are also reported. The effect of treatment is expressed as enlarged vs reduced, sex as female vs. male, and hatching year as 2003 vs 2004 or 2006.

|  | **Overall Recruitment** | | | **Conditional on fledging** | | |
| --- | --- | --- | --- | --- | --- | --- |
| **Fixed effects** | ***Estimate*** | ***Std. Error*** | ***P*** | ***Estimate*** | ***Std. Error*** | ***P*** |
| (Intercept) | -0.96 | 0.46 | - | -0.63 | 0.42 | - |
| Treatment (reduced) | 1.08 | 0.49 | **0.031** | 0.89 | 0.46 | 0.057 |
| Sex (male) | -0.21 | 0.42 | 0.619 | -0.09 | 0.42 | 0.835 |
| Hatching year (2004) | -1.05 | 0.59 | **0.006** | -0.91 | 0.55 | **0.011** |
| Hatching year (2006) | -1.79 | 0.66 | **0.006** | -1.60 | 0.63 | **0.011** |
| **Random effects** | **Variance** | | | **Variance** | | |
| Nest identity | 1.48 | | | 0.98 | | |
| **Sample size** | | | | | | |
| Nests | 89 | | | 85 | | |
| Individuals | 212 | | | 168 | | |

**Table S8.** Results of a generalized linear mixed-effects model testing the relationship between fledging age (in days) and recruitment probability to adulthood. Fledging age was included as both linear and quadratic terms to allow for a non-linear relationship. The model also included brood-size treatment (reduced vs enlarged) and hatching year as fixed effects. Estimates are presented as fixed-effect coefficients with standard errors and P-values obtained from likelihood ratio tests based on single-term deletions from the fitted model. Variance components of the random effect and sample sizes are reported. The effect of treatment is expressed as enlarged vs reduced, and hatching year as 2003 vs 2004 or 2006.

| **Fixed effects** | ***Estimate*** | ***Std. Error*** | ***P*** |
| --- | --- | --- | --- |
| (Intercept) | -168.43 | 73.74 | - |
| Fledging age (linear) | 6.05 | 2.64 | **0.002** |
| Fledging age (quadratic) | -0.05 | 0.02 | **0.002** |
| Treatment (reduced) | 0.68 | 0.49 | 0.166 |
| Hatching year (2004) | -0.87 | 0.59 | 0.101 |
| Hatching year (2006) | -1.19 | 0.66 | 0.101 |
| **Random effects** | **Variance** | | |
| Nest identity | 1.01 | | |
| **Sample size** | | | |
| Nests | 85 | | |
| Individuals | 168 | | |

**Table S9.** Estimated marginal means of morphological traits measured at three developmental stages (50 days of age, fledging age, and adulthood) from Gaussian mixed-effects models. Means are shown for each brood-size manipulation treatment (enlarged, reduced) and recruitment status (recruited, not recruited). Values represent model-adjusted means ± SE, accounting for sex and hatching year as fixed effects and nest identity as a random effect. Pairwise contrasts between brood-size treatments within each recruitment group were assessed using Tukey-adjusted comparisons based on the fitted models.

| **Development stage** | **Trait** | **Recruitment status** | **Enlarged broods** | **Reduced broods** | **Δ (enlarged - reduced)** | **P-value** |
| --- | --- | --- | --- | --- | --- | --- |
| **Day 50** | Body mass (g) | Recruited | 91.3 ± 1.70 | 95.0 ± 2.00 | -3.78 ± 2.61 | 0.148 |
|  |  | Non-recruited | 88.5 ± 1.19 | 93.6 ± 1.63 | -5.04 ± 2.02 | **0.013** |
|  | Wing length (mm) | Recruited | 212 ± 2.34 | 217 ± 2.77 | -5.68 ± 3.58 | 0.113 |
|  |  | Non-recruited | 207 ± 1.68 | 215 ± 2.26 | -8.12 ± 2.82 | **0.004** |
|  | Sternum length (mm) | Recruited | 39.4 ± 0.31 | 40.2 ± 0.34 | -0.81 ± 0.45 | 0.074 |
|  |  | Non-recruited | 38.7 ± 0.19 | 39.6 ± 0.28 | -0.91 ± 0.34 | **0.007** |
| **Fledging age** | Body mass (g) | Recruited | 90.0 ± 1.55 | 93.8 ± 1.71 | -3.79 ± 2.29 | 0.097 |
|  |  | Non-recruited | 96.4 ± 0.98 | 90.7 ± 1.45 | -4.32 ± 1.76 | **0.014** |
|  | Wing length (mm) | Recruited | 222 ± 1.09 | 223 ± 0.94 | -1.02 ± 1.42 | 0.474 |
|  |  | Non-recruited | 220 ± 0.66 | 222 ± 0.91 | -2.39 ± 1.13 | **0.035** |
|  | Sternum length (mm) | Recruited | 39.7 ± 0.24 | 40.3 ± 0.28 | -0.617 ± 0.36 | 0.087 |
|  |  | Non-recruited | 39.3 ± 0.16 | 39.8 ± 0.23 | -0.51 ± 0.29 | 0.072 |
| **Adulthood** | Body mass (g) | Recruited | 98.3 ± 0.92 | 99.5 ± 1.05 | -1.21 ± 1.30 | 0.354 |
|  | Wing length (mm) | Recruited | 227 ± 0.80 | 227 ± 0.88 | -0.13 ± 1.12 | 0.910 |
|  | Sternum length (mm) | Recruited | 40.2 ± 0.28 | 40.7 ± 0.31 | -0.45 ± 0.40 | 0.255 |

**Table S10.** Results of linear mixed models testing the effects of brood size manipulation treatment, sex, and hatching year on the morphometric traits of adult birds reared in these broods. Estimates are presented as fixed-effect coefficients ± SE, with P-values obtained from likelihood ratio tests based on single-term deletions from the fitted models. Variance components of the random effects and sample sizes are also reported. The effect of treatment is expressed as enlarged vs reduced, sex as female vs. male, and hatching year as 2003 vs 2004 or 2006.

|  | **Adult body mass (g)** | | **Adult wing length (mm)** | | **Adult sternum length (mm)** | |
| --- | --- | --- | --- | --- | --- | --- |
| **Fixed effects** | ***Estimate ± SE*** | ***P*** | ***Estimate ± SE*** | ***P*** | ***Estimate ± SE*** | ***P*** |
| (Intercept) | 97.88 ± 2.56 | - | 225.09 ± 1.09 | - | 39.84 ± 0.39 | - |
| Treatment (reduced) | 1.21 ± 1.30 | 0.357 | 0.13 ± 1.12 | 0.910 | 0.45 ± 0.40 | 0.258 |
| Sex (male) | 0.82 ± 1.30 | 0.522 | 2.50 ± 1.09 | **0.025** | 0.77 ± 0.39 | 0.052 |
| Hatching year (2004) | 0.16 ± 1.54 | 0.872 | 1.06 ± 1.34 | 0.722 | -0.24 ± 0.47 | 0.676 |
| Hatching year (2006) | 0.96 ± 1.84 | 0.872 | 0.55 ± 1.51 | 0.722 | -0.24 ± 0.47 | 0.676 |
| Age (centred) | -0.11 ± 0.17 | 0.523 | 0.30 ± 0.04 | **< 0.001** | 0.00 ± 0.02 | 0.982 |
| Time of capture * | 0.47 ± 0.14 | **0.001** | - | - | - | - |
| Day of capture * | -0.13 ± 0.02 | **< 0.001** | - | - | - | - |
| **Random effects** | **Variance** | | **Variance** | | **Variance** | |
| Bird identity | 8.12 | | 12.76 | | 1.61 | |
| Residual | 5.79 | | 1.42 | | 0.46 | |
| **Sample size** | | | | | | |
| Individuals | 47 | | 47 | | 47 | |
| Observations | 202 | | 171 | | 152 | |

* Time of capture (hour within the day) and day of capture (since May 1) were included as additional covariates only in the body mass model.

**Table S11.** Results of generalized linear mixed-effects models testing the association between morphological traits measured on day 50 after hatching (body mass, wing length, and sternum length) and recruitment probability. Separate models were fitted for each trait. Models included brood-size manipulation treatment, sex, and hatching year as fixed effects, with nest identity as a random intercept. Estimates are presented as fixed-effect coefficients ± SE on the logit scale, with P-values obtained from likelihood ratio tests based on single-term deletions from the fitted models. Variance components of the random effects and sample sizes are also reported. The effect of treatment is expressed as enlarged vs reduced, sex as female vs. male, and hatching year as 2003 vs 2004 or 2006.

|  | **Recruitment by mass (gram)** | | **Recruitment by wing length (mm)** | | **Recruitment by sternum length (mm)** | |
| --- | --- | --- | --- | --- | --- | --- |
| **Fixed effects** | ***Estimate ± SE*** | ***P*** | ***Estimate ± SE*** | ***P*** | ***Estimate ± SE*** | ***P*** |
| (Intercept) | -5.95 ± 2.96 | - | -18.00 ± 6.89 | - | -19.48 ± 8.12 | - |
| Size measurement | 0.06 ± 0.03 | **0.041** | 0.08 ± 0.03 | **0.001** | 0.49 ± 0.21 | **0.006** |
| Treatment (reduced) | 0.82 ± 0.49 | 0.097 | 0.54 ± 0.49 | 0.276 | 0.71 ± 0.54 | 0.192 |
| Sex (male) | 0.01 ± 0.43 | 0.977 | -0.14 ± 0.43 | 0.741 | -0.44 ± 0.48 | 0.357 |
| Hatching year (2004) | -1.16 ± 0.59 | **0.045** | -0.67 ± 0.57 | 0.094 | -1.03 ± 0.63 | **0.007** |
| Hatching year (2006) | -1.26 ± 0.68 | **0.045** | -1.26 ± 0.64 | 0.094 | -1.93 ± 0.75 | **0.007** |
| **Random effects** | **Variance** | | **Variance** | | **Variance** | |
| Nest identity | 1.06 | | 0.88 | | 1.50 | |
| **Sample size** | | | | | | |
| Nests | 84 | | 84 | | 84 | |
| Individuals | 167 | | 167 | | 166 | |

**Table S12.** Results of linear models testing the effects of brood-size manipulation treatment, sex, and hatching year on three life-history traits: age at first reproduction, lifespan, and reproductive lifespan. Estimates are presented as fixed-effect coefficients ± SE, with P-values obtained from likelihood ratio tests based on single-term deletions from the fitted models. The effect of treatment is expressed as enlarged vs reduced, sex as female vs. male, and hatching year as 2003 vs 2004 or 2006.

|  | **Age at first reproduction (years)** | | **Lifespan (years)** | | **Reproductive lifespan (years)** | |
| --- | --- | --- | --- | --- | --- | --- |
| **Fixed effects** | ***Estimate ± SE*** | ***P*** | ***Estimate ± SE*** | ***P*** | ***Estimate ± SE*** | ***P*** |
| (Intercept) | 2.52 ± 0.24 | - | 1.63 ± 0.15 | - | 1.18 ± 0.26 | - |
| Treatment (reduced) | 0.05 ± 0.24 | 0.836 | -0.14 ± 0.156 | 0.381 | -0.31 ± 0.27 | 0.250 |
| Sex (male) | 0.35 ± 0.24 | 0.143 | 0.18 ± 0.15 | 0.237 | 0.03 ± 0.26 | 0.921 |
| Hatching year (2004) | 0.34 ± 0.28 | 0.124 | -0.04 ± 0.19 | 0.892 | -0.07 ± 0.31 | 0.958 |
| Hatching year (2006) | 0.65 ± 0.32 | 0.124 | 0.07 ± 0.20 | 0.892 | -0.08 ± 0.35 | 0.958 |
| **Sample size** | | | | | | |
| Individuals | 39 | | 39 | | 39 | |

**Table S13.** Results of a generalized linear model testing the effects of brood-size manipulation treatment, sex, hatching year, and the interaction between treatment and sex on the number of breeding attempts during an individual’s reproductive lifetime. Estimates are presented as fixed-effect coefficients with standard errors and P-values obtained from likelihood ratio tests based on single-term deletions from the fitted models. The effect of treatment is expressed as enlarged vs reduced, sex as female vs. male, and hatching year as 2003 vs 2004 or 2006.

| **Fixed effects** | ***Estimate*** | ***Std. Error*** | ***P*** |
| --- | --- | --- | --- |
| (Intercept) | 1.61 | 0.24 | - |
| Treatment (reduced) | -0.81 | 0.34 | - |
| Sex (male) | -0.73 | 0.33 | - |
| Hatching year (2004) | -0.39 | 0.30 | 0.414 |
| Hatching year (2006) | -0.11 | 0.33 | 0.414 |
| Treatment (reduced): Sex (male) | 1.13 | 0.50 | **0.026** |
| **Sample size** | | | |
| Individuals (M: F) | 42 (19: 23) | | |

**Table S14.** Results of a generalized linear model testing the effects of brood-size manipulation treatment, sex, and hatching year on the annual breeding probability. Estimates are presented as fixed-effect coefficients with standard errors and P-values obtained from likelihood ratio tests based on single-term deletions from the fitted models. The effect of treatment is expressed as enlarged vs reduced, sex as female vs. male, and hatching year as 2003 vs 2004 or 2006.

| **Fixed effects** | ***Estimate*** | ***Std. Error*** | ***P*** |
| --- | --- | --- | --- |
| (Intercept) | 2.07 | 0.44 | - |
| Treatment (reduced) | -0.78 | 0.47 | 0.095 |
| Sex (male) | -0.03 | 0.46 | 0.948 |
| Hatching year (2004) | -0.74 | 0.50 | 0.328 |
| Hatching year (2006) | -0.15 | 0.64 | 0.328 |
| **Sample size** | | | |
| Individuals (M: F) | 39 (16: 23) | | |

**Table S15.** Estimated marginal means for age at first reproduction, lifespan, reproductive lifespan, and number of breeding attempts (breeding years). Estimated marginal means ± SE are shown for each brood-size manipulation treatment (enlarged, reduced) and sex (male, female). Estimates were obtained from Gaussian mixed-effects models including brood-size treatment, sex, their interaction, and hatching year as fixed effects, with nest identity fitted as a random effect. Pairwise contrasts between brood-size treatments within each sex are reported as enlarged/reduced ratios with Tukey-adjusted p-values.

| **Trait** | **Sex** | **Enlarged broods** | **Reduced broods** | **Rate ratio (enlarged/ reduced)** | **P-value** |
| --- | --- | --- | --- | --- | --- |
| **Age at first reproduction** | Males | 3.21 ± 0.26 | 3.24 ± 0.28 | -0.04 ± 0.37 | 0.924 |
|  | Females | 2.84 ± 0.21 | 2.90 ± 0.24 | -0.06 ± 0.32 | 0.851 |
| **Lifespan** | Males | 5.52 ± 0.85 | 6.13 ± 1.00 | 0.90 ± 0.19 | 0.626 |
|  | Females | 5.76 ± 0.80 | 3.94 ± 0.65 | 1.46 ± 0.32 | 0.086 |
| **Reproductive lifespan** | Males | 2.61 ± 0.73 | 2.89 ± 0.88 | 0.90 ± 0.36 | 0.795 |
|  | Females | 3.55 ± 0.80 | 1.92 ± 0.49 | 1.85 ± 0.64 | 0.083 |
| **Breeding attempts** | Males | 2.05 ± 0.54 | 2.83 ± 0.78 | 0.73 ± 0.27 | 0.385 |
|  | Females | 4.24 ± 0.82 | 1.89 ± 0.52 | 2.25 ± 0.76 | **0.017** |

**Table S16.** Results of generalized linear models testing the effects of brood size manipulation treatment, sex, and hatching year on lifetime reproductive success, measured as the total number of eggs, hatchlings, fledglings, and recruits produced by each individual. Estimates are presented as fixed-effect coefficients ± SE with corresponding P-values obtained from likelihood ratio tests based on single-term deletions from the fitted model. Models were fitted using a negative binomial distribution for eggs, hatchlings, and fledglings, and a Poisson distribution for the number of recruits. The interaction between treatment and sex was included when significant and removed otherwise. Sample size corresponds to the number of individuals included in each analysis (males: females). The effect of treatment is expressed as enlarged vs reduced, sex as female vs. male, and hatching year as 2003 vs 2004 or 2006.

|  | **Total number of eggs** | | **Total number of hatchlings** | | **Total number of fledglings** | | **Total number of recruits** | |
| --- | --- | --- | --- | --- | --- | --- | --- | --- |
| **Fixed effects** | ***Estimate ± SE*** | ***P*** | ***Estimate ± SE*** | ***P*** | ***Estimate ± SE*** | ***P*** | ***Estimate ± SE*** | ***P*** |
| (Intercept) | 2.56 ± 0.24 | - | 2.03 ± 0.24 | - | 1.61 ± 0.27 | - | -0.76 ± 0.55 | - |
| Treatment (reduced) | -0.82 ± 0.32 | - | -0.60 ± 0.26 | **0.025** | -0.46 ± 0.28 | 0.119 | -0.54 ± 0.64 | 0.383 |
| Sex (male) | -0.68 ± 0.31 | - | -0.42 ± 0.25 | 0.099 | -0.44 ± 0.265 | 0.105 | -1.41 ± 0.78 | **0.039** |
| Hatching year (2004) | -0.40 ± 0.28 | 0.353 | -0.13 ± 0.30 | 0.547 | 0.02 ± 0.33 | 0.211 | -0.14 ± 0.76 | 0.546 |
| Hatching year (2006) | -0.01 ± 0.30 | 0.353 | 0.25 ± 0.32 | 0.547 | 0.55 ± 0.33 | 0.211 | 0.65 ± 0.67 | 0.546 |
| Treatment (reduced): Sex (male) | 1.18 ± 0.46 | **0.013** | - | - | - | - | - | - |
| **Sample size** | | | | | | | | |
| Individuals (M: F) | 42 (19: 23) | | 42 (19: 23) | | 42 (19: 23) | | 42 (19: 23) | |

**Table S17.** Results of generalized linear models testing the effects of brood-size manipulation treatment, sex, and hatching year on annual reproductive success, measured as the number of eggs and hatchlings produced per breeding attempt. The number of breeding attempts was included as an offset to account for variation in reproductive lifespan. Estimates are presented as fixed-effect coefficients ± SE with corresponding P-values obtained from likelihood ratio tests based on single-term deletions from the fitted models. The effect of treatment is expressed as enlarged vs reduced, sex as female vs. male, and hatching year as 2003 vs 2004 or 2006.

|  | **Number of eggs per year** | | **Number of hatchlings per year** | |
| --- | --- | --- | --- | --- |
| **Fixed effects** | ***Estimate ± SE*** | ***P*** | ***Estimate ± SE*** | ***P*** |
| (Intercept) | 0.90 ± 0.04 | - | 0.57 ± 0.13 | - |
| Treatment (reduced) | 0.03 ± 0.04 | 0.489 | -0.27 ± 0.15 | 0.085 |
| Sex (male) | 0.08 ± 0.04 | 0.053 | -0.13 ± 0.14 | 0.382 |
| Hatching year (2004) | 0.04 ± 0.05 | **0.042** | 0.16 ± 0.17 | 0.021 |
| Hatching year (2006) | 0.14 ± 0.05 | **0.042** | 0.49 ± 0.16 | 0.021 |
| **Sample size** | | | | |
| Individuals (M: F) | 42 (19: 23) | | 42 (19: 23) | |

**Table S18.** Results of mixed-effects models testing the effects of brood-size manipulation treatment, sex, hatching year, and age on age-specific reproductive performance. Count responses (clutch size, number of hatchlings, and number of fledglings) were modelled using a Conway–Maxwell–Poisson distribution, whereas rate responses (hatching rate and fledging rate) were modelled using a beta-binomial distribution. Estimates are presented as fixed-effect coefficients ± SE with corresponding P-values obtained from likelihood ratio tests based on single-term deletions from the fitted models. Age was decomposed into within-individual (age centred on each individual’s mean age) and between-individual (mean age of each individual across observations) components, both of which were standardised prior to analysis. Variance components of the random effects and sample sizes are also reported. The effect of treatment is expressed as enlarged vs reduced, sex as female vs. male, and hatching year as 2003 vs 2004 or 2006.

|  | **Clutch size** | | **Number of Hatchlings** | | **Hatching rate** | | **Number of fledglings** | | **Fledging rate** | |
| --- | --- | --- | --- | --- | --- | --- | --- | --- | --- | --- |
| **Fixed effects** | ***Estimate ± SE*** | ***P*** | ***Estimate ± SE*** | ***P*** | ***Estimate ± SE*** | ***P*** | ***Estimate ± SE*** | ***P*** | ***Estimate ± SE*** | ***P*** |
| (Intercept) | 0.95 ± 0.04 | - | 0.10 ± 0.15 | - | 0.23 ± 0.36 | - | -0.60 ± 0.27 | - | -0.26 ± 0.41 | - |
| Treatment (enlarged) | -0.04 ± 0.05 | 0.405 | 0.43 ± 0.15 | - | 0.75 ± 0.37 | - | 0.39 ± 0.28 | - | -0.06 ± 0.46 | - |
| Sex (male) | 0.05 ± 0.04 | 0.267 | -0.03 ± 0.13 | 0.789 | -0.69 ± 0.36 | 0.057 | 0.07 ± 0.23 | 0.769 | 0.28 ± 0.41 | 0.493 |
| Age (within) | 0.01 ± 0.02 | 0.594 | -0.26 ± 0.15 | - | -0.35 ± 0.26 | - | -0.09 ± 0.13 | 0.492 | -0.43 ± 0.21 | **0.039** |
| Age (between) | -0.02 ± 0.02 | 0.304 | -0.45 ± 0.13 | - | -0.42 ± 0.17 | **0.015** | -0.87 ± 0.24 | - | -1.00 ± 0.41 | - |
| Hatching year (2004) | 0.03 ± 0.05 | 0.066 | 0.12 ± 0.15 | **0.011** | 0.28 ± 0.42 | **0.005** | 0.31 ± 0.26 | 0.444 | 0.59 ± 0.49 | 0.052 |
| Hatching year (2006) | 0.14 ± 0.06 | 0.066 | 0.47 ± 0.15 | **0.011** | 1.77 ± 0.61 | **0.005** | 0.01 ± 0.31 | 0.444 | -0.77 ± 0.54 | 0.052 |
| Treatment (enlarged): Age (within) | - | - | 0.43 ± 0.16 | **0.009** | 0.86 ± 0.39 | **0.014** | - | - | - | - |
| Treatment (enlarged): Age (between) | - | - | 0.34 ± 0.16 | **0.030** | - | - | 0.84 ± 0.28 | **0.002** | 1.21 ± 0.52 | **0.015** |
| **Random effects** | **Variance** | | **Variance** | | **Variance** | | **Variance** | | **Variance** | |
| Bird identity | 0.00 | | 0.00 | | 0.00 | | 0.00 | | 0.00 | |
| **Sample size** | | | | | | | | | | |
| Individuals | 42 | | 42 | | 42 | | 42 | | 42 | |
| Observations | 125 | | 127 | | 125 | | 127 | | 127 | |

**Table S19.** Results of mixed-effects models testing the effects of brood-size manipulation treatment, environmental quality, sex, age, and hatching year on annual reproductive performance, measured as clutch size, number of hatchlings, hatching rate, number of fledglings, and fledging rate per breeding attempt. Environmental quality was quantified as a colony-wide annual index based on mean nestling survival from hatching to fledging. Estimates are presented as fixed-effect coefficients ± SE with corresponding P-values obtained from likelihood ratio tests based on single-term deletions from the fitted models. Age was standardized prior to analysis. Count responses (clutch size, number of hatchlings, and number of fledglings) were modelled using a Conway–Maxwell–Poisson distribution, whereas rate responses (hatching rate and fledging rate) were modelled using a beta-binomial distribution. Variance components of the random effects and sample sizes are also reported. The effect of treatment is expressed as enlarged vs reduced, sex as female vs. male, and hatching year as 2003 vs 2004 or 2006.

|  | **Clutch size** | | **Number of Hatchlings** | | **Hatching rate** | | **Number of fledglings** | | **Fledging rate** | |
| --- | --- | --- | --- | --- | --- | --- | --- | --- | --- | --- |
| **Fixed effects** | ***Estimate ± SE*** | ***P*** | ***Estimate ± SE*** | ***P*** | ***Estimate ± SE*** | ***P*** | ***Estimate ± SE*** | ***P*** | ***Estimate ± SE*** | ***P*** |
| (Intercept) | 0.94 ± 0.04 | - | 0.24 ± 0.13 | - | 0.36 ± 0.36 | - | -0.29 ± 0.23 | - | 0.07 ± 0.39 | - |
| Treatment (enlarged) | -0.05 ± 0.05 | - | 0.34 ± 0.13 | - | 0.98 ± 0.39 | - | 0.11 ± 0.23 | - | -0.24 ± 0.43 | - |
| Year quality | -0.04 ± 0.03 | - | -0.00 ± 0.10 | - | 0. 08 ± 0.24 | - | 0.25 ± 0.20 | - | 0.42 ± 0.31 | **-** |
| Sex (male) | 0.05 ± 0.04 | 0.269 | -0.12 ± 0.13 | 0.349 | -0.71 ± 0.37 | 0.052 | -0.07 ± 0.22 | 0.751 | 0.02 ± 0.41 | 0.968 |
| Age (within) | 0.03 ± 0.02 | 0.220 | 0.06 ± 0.07 | 0.363 | -0.03 ± 0.18 | 0.864 | -0.21 ± 0.13 | 0.107 | -0.54 ± 0.24 | **0.014** |
| Age (between) | -0.01 ± 0.02 | 0.639 | -0.23 ± 0.07 | **< 0.001** | -0.51 ± 0.18 | **0.004** | -0.42 ± 0.12 | 0.002 | -0.38 ± 0.23 | 0.122 |
| Hatching year (2004) | 0.05 ± 0.05 | **0.016** | 0.12 ± 0.15 | **0.014** | 0.04 ± 0.42 | 0.075 | 0.34 ± 0.26 | 0.348 | 0.67 ± 0.53 | 0.056 |
| Hatching year (2006) | 0.18 ± 0.06 | **0.016** | 0.47 ± 0.16 | **0.014** | 1.30 ± 0.64 | 0.075 | -0.03 ± 0.31 | 0.348 | -0.71 ± 0.54 | 0.056 |
| Treatment (enlarged): Year quality | 0.00 ± 0.03 | 0.914 | 0.13 ± 0.12 | 0.271 | 0.42 ± 0.28 | 0.143 | 0.16 ± 0.24 | 0.498 | 0.00 ± 0.35 | 0.990 |
| **Random effects** | **Variance** | | **Variance** | | **Variance** | | **Variance** | | **Variance** | |
| Bird identity | 0.00 | | 0.00 | | 0.00 | | 0.00 | | 0.03 | |
| **Sample size** | | | | | | | | | | |
| Individuals | 42 | | 42 | | 42 | | 42 | | 42 | |
| Observations | 125 | | 127 | | 125 | | 127 | | 127 | |

**Figure S1.** Model predicted difference in body mass (g; A), wing length (mm; B), and sternum length (mm; C), with 95% CI, between Alpine swift nestlings reared in experimentally enlarged and reduced broods, in relation to age.

**Figure S2.** Predicted probability of recruitment of Alpine swift nestlings as a function of fledging age. The solid line represents the fitted model estimate, and the shaded band indicates the 95% confidence interval. Recruitment probability increases to a peak at intermediate fledging ages and declines sharply at older ages.

**Figure S3.** Relationship between size measured at fledging and during adulthood for (A) mass, (B) wing length, and (C) sternum length of Alpine swifts reared in experimentally enlarged (red) and reduced (blue) broods. Solid grey lines show linear regressions with 95% confidence intervals, and dashed lines indicate the 1:1 relationship between fledging and adult values. Pearson correlation coefficients, 95% confidence intervals, and sample sizes are shown within each panel.

**Figure S4.** Variation in age at first reproduction (A), lifespan (B), and reproductive lifespan (C) of Alpine swifts reared in experimentally enlarged (red) and reduced (blue) broods and recruited as adults in the population. The plot shows means and 95% confidence intervals by sex and natal treatment.

**Figure S5.** Within- and between-individual effects of age on annual reproductive output. Panels (A–B) show within-individual effects, where age is expressed relative to each individual’s mean age, and panels (C–D) show between-individual effects, where age represents the individual’s mean age across observations. Responses are annual number of hatchlings (A, C) and number of fledglings (B, D). Solid lines represent females and dashed lines represent males; colours indicate brood treatment (red = enlarged, blue = reduced) and shaded areas show 95% confidence intervals.

**Figure S6.** Relationship between annual environmental quality and reproductive performance. (A) Annual environmental quality index, calculated as the standardised colony-wide mean nestling survival from hatching to fledging for each year. (B–F) Predicted reproductive output as a function of the year-quality index for individuals from reduced (blue) and enlarged (red) brood treatments. Panels show predicted clutch size (B), number of hatchlings (C), hatching rate (D), number of fledglings (E), and fledging rate (F). Lines represent model-predicted values from mixed-effects models including treatment by environmental quality interactions, with shaded areas indicating 95% confidence intervals; colours indicate brood treatment (red = enlarged, blue = reduced).
